# Overexpression of four MiTFL1 genes from mango delays the flowering time in transgenic Arabidopsis

**DOI:** 10.1186/s12870-021-03199-9

**Published:** 2021-09-07

**Authors:** Yi-Han Wang, Xin-Hua He, Hai-Xia Yu, Xiao Mo, Yan Fan, Zhi-Yi Fan, Xiao-Jie Xie, Yuan Liu, Cong Luo

**Affiliations:** College of Agriculture, State Key Laboratory for Conservation and Utilization of Subtropical Agro-Bioresources, Guangxi University, Nanning, 530004 Guangxi China

**Keywords:** *Mangifera indica* L., *MiTFL1* genes, Expression, Function, Protein interactions

## Abstract

**Background:**

*TERMINAL FLOWER 1* (*TFL1*) belongs to the phosphatidylethanolamine-binding protein (PEBP) family, which is involved in inflorescence meristem development and represses flowering in several plant species. In the present study, four *TFL1* genes were cloned from the mango (*Mangifera indica* L.) variety ‘SiJiMi’ and named *MiTFL1-1*, *MiTFL1-2*, *MiTFL1-3* and *MiTFL1-4*.

**Results:**

Sequence analysis showed that the encoded MiTFL1 proteins contained a conserved PEBP domain and belonged to the TFL1 group. Expression analysis showed that the *MiTFL1* genes were expressed in not only vegetative organs but also reproductive organs and that the expression levels were related to floral development. Overexpression of the four *MiTFL1* genes delayed flowering in transgenic *Arabidopsis*. Additionally, *MiTFL1-1* and *MiTFL1-3* changed the flower morphology in some transgenic plants. Yeast two-hybrid (Y2H) analysis showed that several stress-related proteins interacted with MiTFL1 proteins.

**Conclusions:**

The four *MiTFL1* genes exhibited a similar expression pattern, and overexpression in *Arabidopsis* resulted in delayed flowering. Additionally, *MiTFL1-1* and *MiTFL1-3* overexpression affected floral organ development. Furthermore, the MiTFL1 proteins could interact with bHLH and 14*-*3-3 proteins. These results indicate that the *MiTFL1* genes may play an important role in the flowering process in mango.

**Supplementary Information:**

The online version contains supplementary material available at 10.1186/s12870-021-03199-9.

## Background

Floral transition is an important stage in the life cycle of higher plants. The process underlying the floral transition from the vegetative to reproductive phase is regulated by complex internal signals and external environmental factors [1, 2]. Various flowering response pathways, such as the photoperiod, vernalization, gibberellin, autonomous, ambient temperature, and age-related pathways, have been identified in the model plant *Arabidopsis thaliana* [3]. Numerous genes play essential roles in these processes, and the involved interactions specify the meristem fate [4]. *CONSTANS* (*CO*) contains a zinc finger structure and CCT domain, which activates the transcription of the *FLOWERING LOCUS T* (*FT*) gene by binding to the *FT* promoter region, and the *FT* protein moves from the leaf tissue to the stem apex to initiate the transition of the plant from vegetative to reproductive growth [5, 6]. The *FLOWER LOCUS C* (*FLC*) gene plays a central role in vernalization—the induction of plant flowering [7, 8]—and inhibits flowering by binding to *FT*, *FLOWERING LOCUS D* (*FD*) and *SUPPRESSOR OF OVER-EXPRESSION OF CONSTANS1* (*SOC1*) and inhibiting the expression of these genes [9]. The flower meristem-specific genes *LEAFY* (*LFY*) and *APETALA1* (*AP1*) directly induce shoot apical meristem differentiation, which promotes the entry of plants into the flowering stage, and then, these genes are activated by *FT* or *SOC1* [10].

*TERMINAL FLOWER 1* (*TFL1*), which belongs to the phosphatidylethanolamine-binding protein (PEBP) family, was first identified in *Arabidopsis* [11]. *TFL1* and *FT* are genes with highly homologous sequences but opposite functions. *TFL1* encodes proteins with conserved His88 and Asp144 residues and the typical amino acid triad modules EYD, YFG, and END [12]. The FT protein does not have this structure, a critical reason for the opposite functions of these two genes [13]. *TFL1* genes, as flowering repressors, determine the timing of the transition of the apical meristem into an inflorescence meristem and the branching pattern of the inflorescence [14]. In *Arabidopsis*, the *AtTFL1* gene not only maintains the infinite growth of the stem apical meristem and inflorescence meristem but is also involved in flower formation [15]. In most fruit trees, the function of *TFL1* homologous genes is to delay flowering, similar to the function of *AtTFL1*. For example, the function of *PmTFL1* (from *Prunus mume*) is to delay flowering, as shown in transformed *Arabidopsis* [16]. The antagonistic effect between *FT* and *TFL1* exhibits a certain relationship with competition with *FD* [17, 18]. The *TFL1* gene inhibits the expression of *LFY* and *AP1*, which are downstream of the *FT* gene, by binding to *FD* and inhibiting flowering [19]. Additionally, *LFY* and *AP1* regulate the expression of the *TFL1* gene in an opposite manner: *LFY* serves as an activator, *AP1* is a suppressor, and these two genes form an unclear feedback loop. The flowering of plants depends on the ratio of *TFL1* to *LFY* gene expression. A high ratio maintains the plants in a flowering inhibition period, whereas plants with a low ratio are in the early flowering period [20].

Compared with annual plants, woody fruit trees have a longer juvenile period, which severely affects breeding. Several studies have shown that the overexpression or silencing of flowering-related genes can shorten the juvenile period and promote flowering. For example, the *BpMADS4* gene from silver birch is constitutively overexpressed in apple, and transgenic plants exhibit markedly shortened juvenile and flowering periods [21]. *PcTFL1-1* and *PcTFL1-2* were silenced in European pear using RNAi technology, and the plants showed early flowering traits and a shortened juvenile period [22].

Mango (*Mangifera indica* L.) is a world-famous woody fruit tree that is widely grown in tropical and subtropical areas. Several environmental factors affect mango flowering, including low temperature, water stress, and carbohydrates. Exogenous spraying of potassium nitrate, paclobutrazol and ethephon can promote flowering, whereas spraying of gibberellin inhibits flowering [23]. The publication of the mango genome has provided valuable information regarding the mining of flowering genes [24]. In recent years, several flowering-regulating genes have been isolated and functionally identified in mango, including the flowering-promoting gene *MiSOC1* [25], two *MiAP1* genes [26], three *MiFT* genes [27], and a flowering-suppressing gene (*MiCO*) [28]. The function of *MiTFL1* has not been identified in this plant. In the present study, four *MiTFL1* homologous genes, namely, *MiTFT1-1*, *MiTFT1-2*, *MiTFT1-3* and *MiTFT1-4*, were cloned from *M. indica* L. cv. ‘SiJiMi’. The expression patterns of the four *MiTFL1* genes in different tissues and at different flowering development stages were evaluated. *MiTFL1* gene overexpression vectors were constructed, and the functions of these genes were determined by transformation in *Arabidopsis*. Proteins interacting with MiTFL1 proteins were screened through yeast two-hybrid (Y2H) assays. The results suggest that these four *MiTFL1* genes inhibit mango flowering.

## Results

### Isolation and sequence analysis of *MiTFL1* genes

**Fig. 1:**
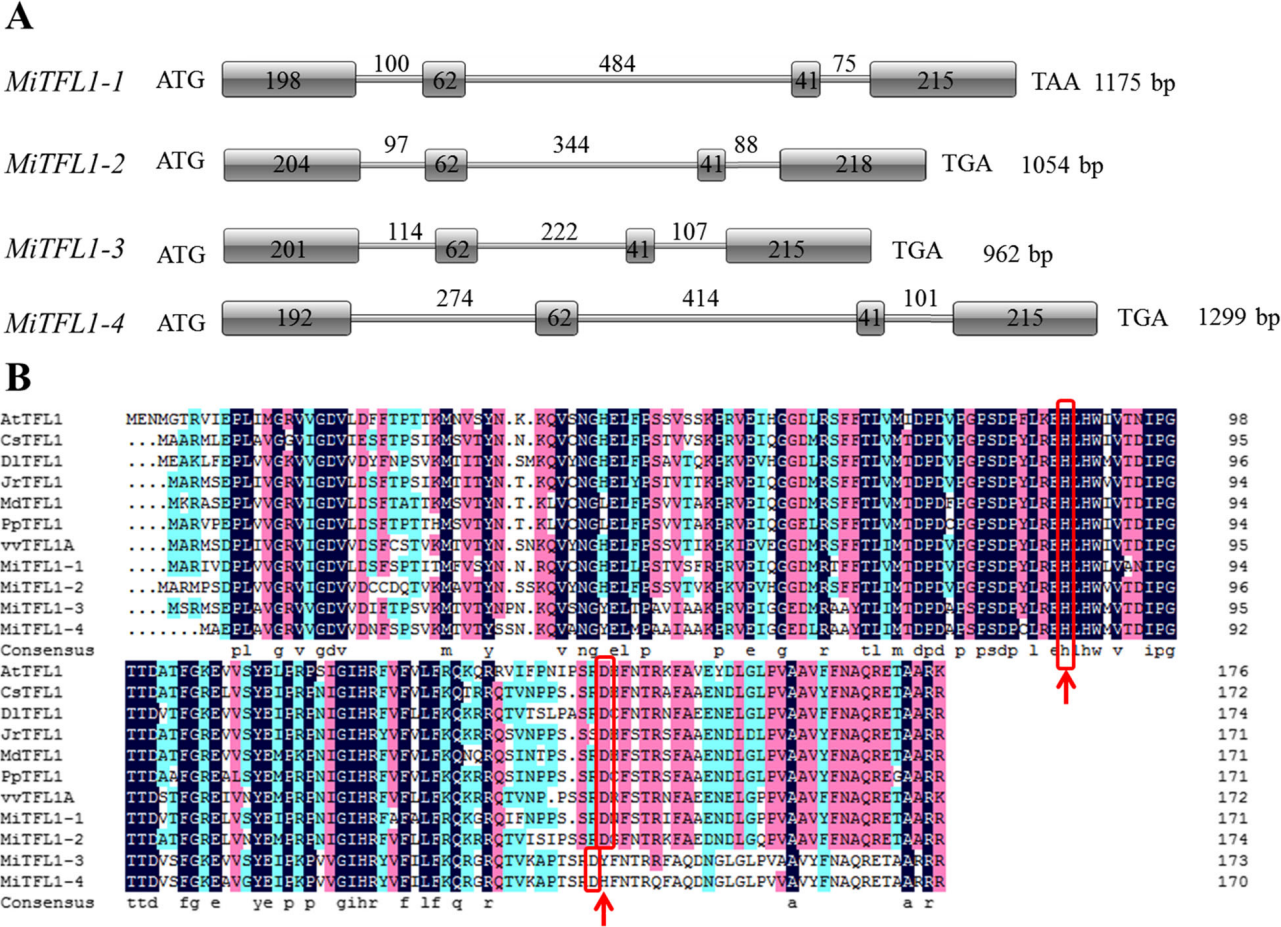
Multiple sequence alignment and gene structure of *TFL1* genes. **a** Gene structures of *MiTFL1* genes. **b** Amino acid sequence alignments of TFL1 proteins from different fruit trees and *Arabidopsis thaliana*. The following species were included in the analysis (the GenBank accession numbers are shown in parentheses): *Arabidopsis thaliana* (AtTFL1, NP_196004.1), *Citrus sinensis* (CsTFL1, NP_001275848), *Dimocarpus longan* (DlTFL1, AHY24028.1), *Juglans regia* (JrTFL1, XP_018811176.1), *Malus domestica* (MdTFL1, NP_001280887.1), *Pyrus x bretschneideri* (PpTFL1, NP_001289244.1), and *Vitis vinifera* (VvTFL1A, NP_001267929.1). The black color indicates that the sequences are exactly the same. The red color indicates ≥75% similarity. The blue color indicates ≥50% similarity. The red box indicates the key residues His85 and Asp140 of TFL1-like proteins

Four *TFL1* homologous genes were identified from the transcriptome and genomic data of ‘SiJiMi’ mango (unpublished data). We further verified the sequences by RT-PCR and showed that these sequences were consistent with those obtained from the transcriptome data. The four genes were named *MiTFL1-1*, *MiTFL1-2*, *MiTFL1-3* and *MiTFL1-4*, and their DNA sequence lengths were 1175 bp, 1054 bp, 962 bp and 1299 bp, respectively. All four *MiTFL1* genes contained four exons and three introns (Fig. 1a). Sequence alignment analysis showed that the length of the first exon differed among the four *MiTFL1* genes, while the second and third exons had the same length; the fourth exon was 215 bp in length in all the genes except *MiTFL1-2*, in which the length was 218 bp. The full coding sequences of the four *MiTFL1* genes were 516 bp, 525 bp, 519 bp and 510 bp and encoded 172 aa, 175 aa, 173 aa and 170 aa, respectively. Amino acid sequence alignment analysis showed that the similarity between MiTFL1-3 and MiTFL1-4 was 90% higher than that between MiTFL1-1 and MiTFL1-2 at 70%. The amino acid sequences of the MiTFL1-1, MiTFL1-2, MiTFL1-3, and MiTFL1-4 proteins exhibited 68.9, 69.1, 62.9 and 60.1% similarity with the sequence of AtTFL1 (NP_196004.1) of *Arabidopsis*, respectively. Additionally, all the MiTFL1 proteins were identified as TFL1 proteins containing the crucial conserved amino acid residues of TFL1-like proteins (Fig. 1b), namely, histidine at position 85 (H85) and aspartic acid at position 140 (D140).

**Fig. 2:**
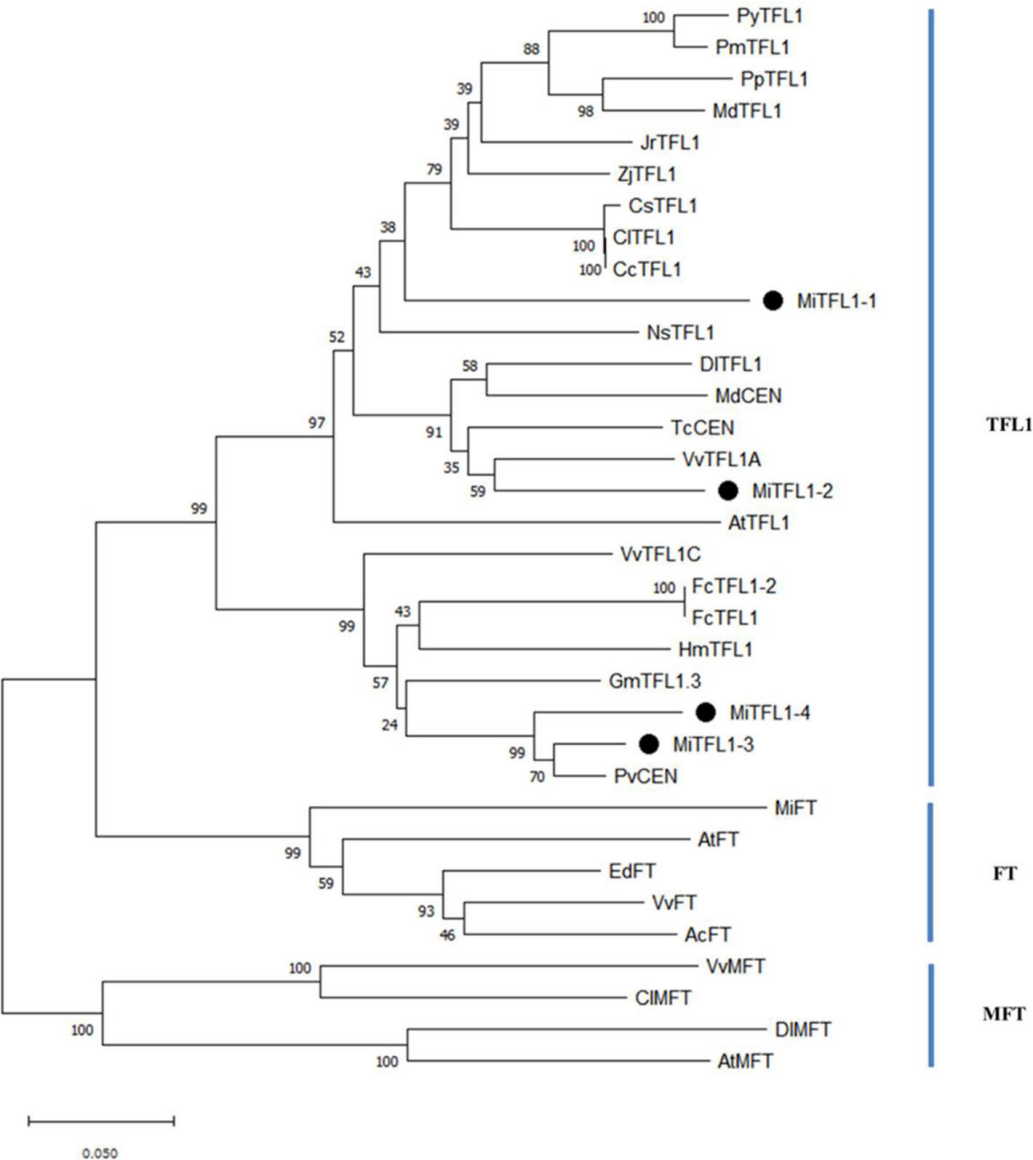
Phylogenetic tree analysis of PEBP proteins. The following species were included in the analysis (the GenBank accession numbers are shown in parentheses): *Arabidopsis thaliana* (AtTFL1, NP_196004.1), *Citrus sinensis* (CsTFL1, NP_001275848), *Dimocarpus longan* (DlTFL1, AHY24028.1), *Juglans regia* (JrTFL1, XP_018811176.1), *Malus domestica* (MdTFL1, NP_001280887.1), *Pyrus x bretschneideri* (PpTFL1, NP_001289244.1), *Malus domestica* (MdCEN, NP_001280940.1), *Pistacia vera* (PvCEN protein 1, XP_031269481.1), *Theobroma cacao* (TcCEN, XP_017973069.1), *Hydrangea macrophylla* (HmTFL1, MF374628.1), *Glycine max* (GmTFL1.3, FJ573238.1), *Ficus carica* (FcTFL1-2, AB746842.1), *Citrus clementina* (CcTFL1, XP_006430226.1), *Citrus limon* (ClTFL1, AWW25018.1), *Ficus carica* (FcTFL1, BAX00857.1), *Nicotiana sylvestris* (NsTFL1, XP_009766168.1), *Prunus mume* (PmTFL1, AEO72021.1), *Prunus yedoensis* (PyTFL1, AEO72023.1), *Vitis vinifera* (VvTFL1A, NP_001267929.1), *Vitis vinifera* (VvTFL1C, NP_001267933.1), *Ziziphus jujube* (ZjTFL1, XP_015898753.1), *Actinidia chinensis* (AcFT, AJA40932.1), *Arabidopsis thaliana* (AtFT, BAA77838.1), *Eriobotrya deflexa* (EdFT, AMB72867.1), *Mangifera indica* (MiFT, AGA19021.1), *Vitis vinifera* (VvFT, NP_001267907.1), *Arabidopsis thaliana* (AtMFT, OAP13671.1), *Citrus limon* (ClMFT, AWW25016.1), *Dimocarpus longan* (DlMFT, AUG98253.1), and *Vitis vinifera* (VvMFT, NP_001267935.1)

The PEBP gene family is divided into the *TFL1*, *FT* and *MFT* subfamilies. According to phylogenetic tree analysis (Fig. 2), the MiTFL1-1, MiTFL1-2, MiTFL1-3 and MiTFL1-4 proteins were clustered with the TFL1 proteins of other species. Among the investigated proteins, the MiTFL1-1 protein is closely related to the TFL1 proteins of apple, pear, apricot, plum, walnut, jujube and other fruit trees of Rutaceae and rose plants. The MiTFL1-2 protein is closely related to the TFL1 proteins of longan and grape and the CEN proteins of apple and cocoa. The MiTFL1-3 and MiTFL1-4 proteins are clustered together and closely related to the PvCEN protein of pistachio.

### Expression analysis of *MiTFL1* genes

**Fig. 3:**
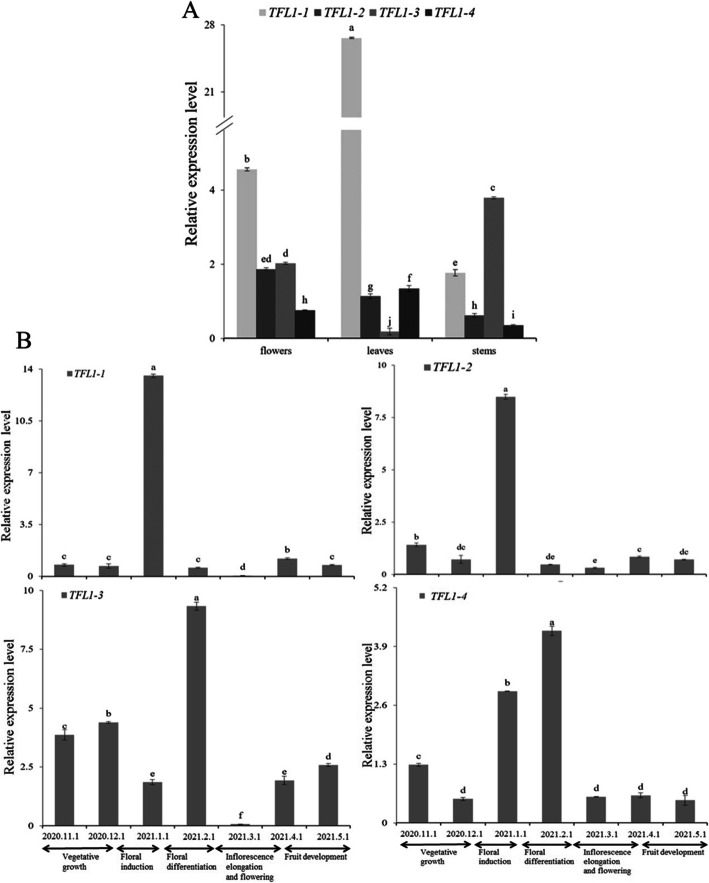
qRT-PCR analysis of the expression profiles of *MiTFL1* genes in mango. **A**: Expression patterns in different tissues. **B**: Expression patterns in different flowering developmental stages. The significance of the differences among the samples was assessed by Student’s t-test (*P* < 0.05). **A** Expression pattern of *MiTFL1* genes in various tissues (flowers, leaves, and stems). **B** Expression pattern of *MiTFL1* genes in mature leaves over time

The expression pattern of *MiTFL1* genes in different mango tissues, including flowers, leaves, and stems, was determined by qRT-PCR (Fig. 3A). *MiTFL1-1*, *MiTFL1-2*, *MiTFL1-3* and *MiTFL1-4* were expressed in flowers, leaves, and stems. *MiTFL1-1* and *MiTFL1-4* exhibited higher expression levels in leaves than in other tissues. *MiTFL1-2* was highly expressed in flowers, while *MiTFL1-3* was highly expressed in stems.

To explore the expression patterns of the *MiTFL1* genes at different flowering development stages of mango, mature leaves of *M. indica* L*.* cv*.* ‘SiJiMi’ were collected from the vegetative growth period to the flowering and fruiting period (November 2020–May 2021), and the results from the analysis of these leaves are shown in Fig. 3B. The expression patterns of the *MiTFL1-1* and *MiTFL1-2* genes were similar. The expression levels of these two genes were significantly higher at the floral induction stage than at other stages. The expression level of the *MiTFL1-3* gene was high during vegetative growth, decreased significantly during the floral induction period, and increased significantly during the floral differentiation period. The lowest expression level was found at the flowering stage, and a relatively high expression level in leaves was observed during fruit development. The expression level of *MiTFL1-4* first decreased during the floral induction period and then increased, peaking during the floral differentiation period, which was followed by a decrease and eventually stabilization during the fruit development period.

### Subcellular localization of MiTFL1 proteins

**Fig. 4:**
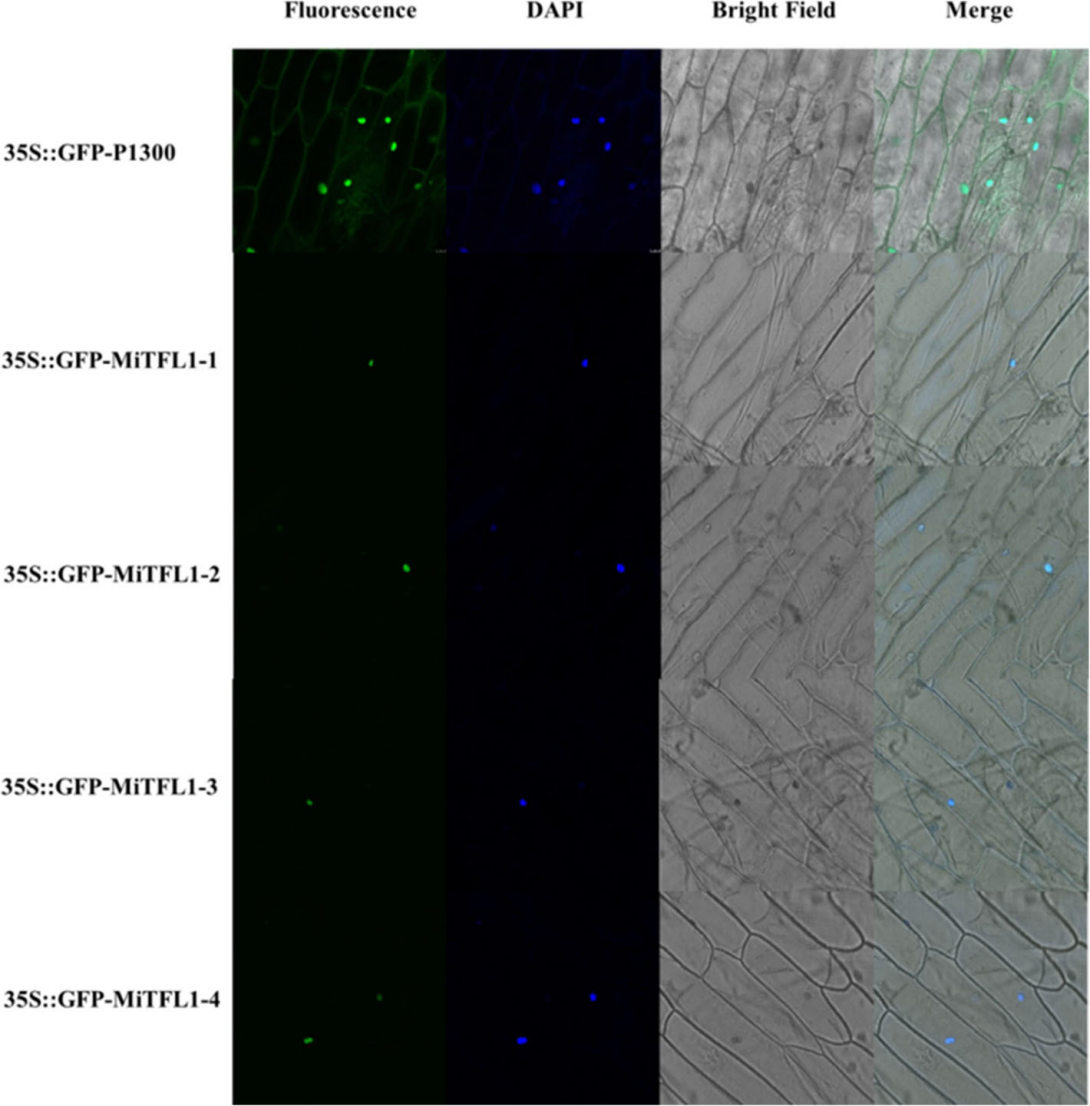
Subcellular localization analysis of MiTFL1 proteins. 35S::GFP-P1300 and 35S::GFP- MiTFL1s were localized in onion epidermal cells

To examine the subcellular localization of MiTFL1 proteins, 35S::GFP-MiTFL1-1, 35S::GFP-MiTFL1-2, 35S::GFP-MiTFL1-3, 35S::GFP- MiTFL1-4 and 35S::GFP-P1300 were separately transformed into onion epidermal cells (Fig. 4). The fluorescence signal of the empty vector 35S::GFP-P1300 was observed in the entire cell. The 35S::GFP-MiTFL1-1, 35S::GFP-MiTFL1-2, 35S::GFP-MiTFL1-3, and 35S::GFP-MiTFL1-4 fusion proteins were visible in only the nucleus and were stained with DAPI.

### Phenotypic analysis of *MiTFL1* overexpression in *Arabidopsis thaliana*

#### *MiTFL1* genes delay flowering in *Arabidopsis thaliana*

To explore the function of *MiTFL1-1*, *MiTFL1-2*, *MiTFL1-3* and *MiTFL1-4* in the flowering process of mango, individual overexpression vectors of pBI121-MiTFL1 were constructed and transferred separately into WT *A. thaliana*. Phenotypic observations of T3-generation homozygous plants were conducted, and WT and pBI121 empty vector-expressing *Arabidopsis* served as controls.

**Fig. 5:**
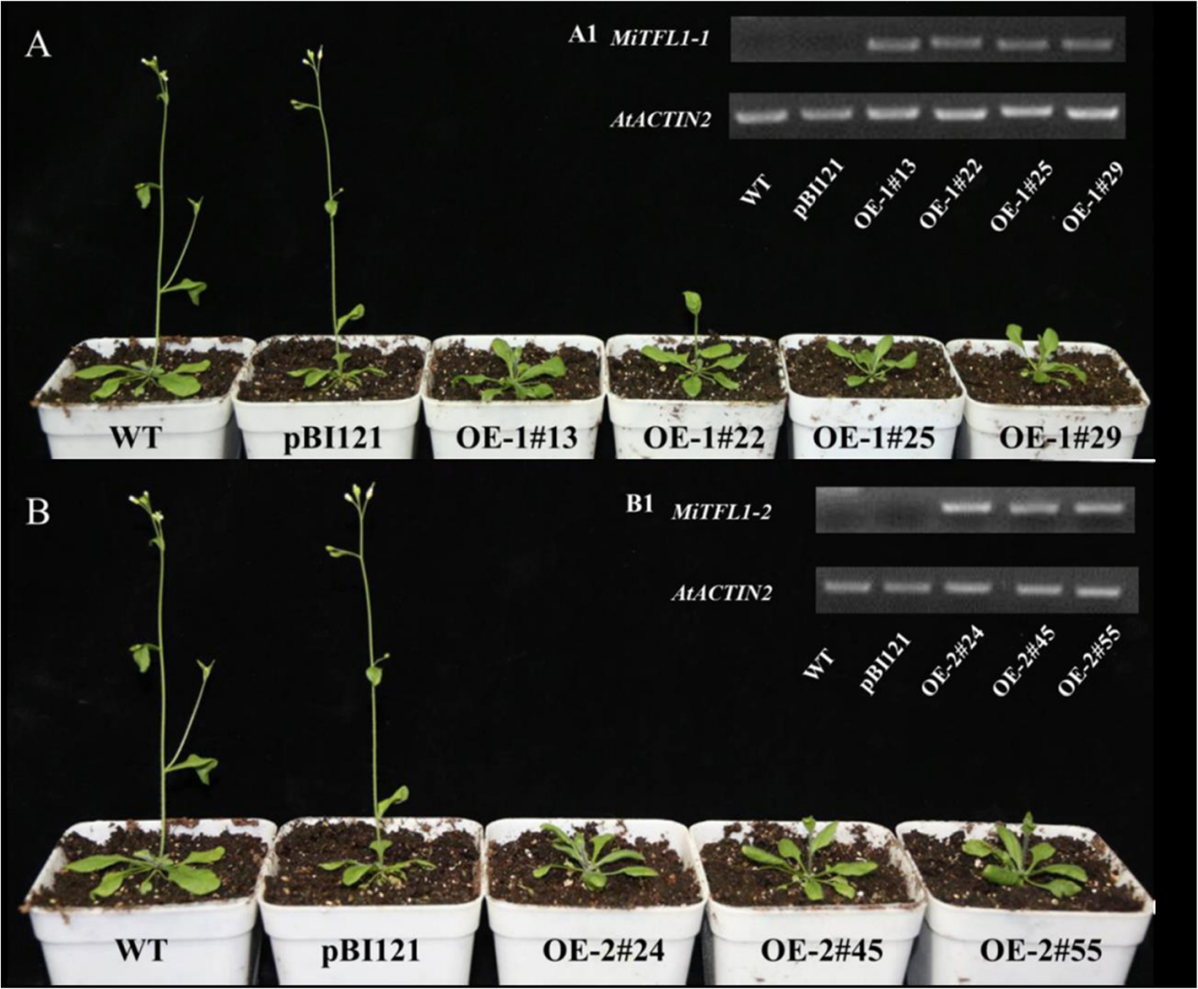
Phenotype of transgenic *Arabidopsis* lines and expression profiles of the transgenes. **A** Phenotype of MiTFL1-1-overexpressing transgenic lines showing delayed flowering (right) and that of the WT and pBI121 transgenic lines as controls (left) under LD conditions. **A1** RT-PCR analysis of *MiTFL1-1* in the control and *MiTFL1-1* overexpression transgenic lines. **B** Phenotype of *MiTFL1-2* overexpression transgenic lines showing delayed flowering (right) and that of the WT and pBI121 transgenic lines as controls (left) under LD conditions. **B1** RT-PCR analysis of *MiTFL1-2* in the control and MiTFL1-2-overexpressing transgenic lines. The original data can be viewed in Fig. S1a-b

Four independent lines with *MiTFL1-1* overexpression (OE-1#13, OE-1#22, OE-1#25 and OE-1#29) and three independent lines with *MiTFL1-2* overexpression (OE-2#24, OE-2#45 and OE-2#55) were selected for functional analysis. Semiquantitative RT-PCR analysis showed that *MiTFL1-1* and *MiTFL1-2* were abundantly expressed in the transgenic lines but absent in the empty vector-expressing transgenic or WT plants (Fig. 5A1 and B1). All the independent lines of *MiTFL1-1* and *MiTFL1-2* showed delayed bolting and flowering: these processes occurred at 28.7–32.5 and 33.3–42.2 days, respectively, in these lines and at 24.9–25.3 and 28.5–28.8 days, respectively, in the control plants (Fig. 5A and B and Table 1). All the transformant lines with *MiTFL1-1* and *MiTFL1-2* showed normal bolting similar to that observed in the WT plants. Additionally, compared with the heights of the WT plants, those of the *MiTFL1-1* and *MiTFL1-2* plants were significantly increased, and the rosette leaves were not significantly affected except in OE-1#13, OE-1#29 and OE-2#45 (Table 1).

**Table 1:** Flowering phenotype analysis of WT, pBI121, MiTFL1-1-overexpressing (OE-1) and MiTFL1-2-overexpressing (OE-2) transgenic plants

ID	Number	Days to bolting (d)	Days to flowering (d)	No. of rosette leaves	Plant height (cm)
WT	13	25.3 ± 0.1	28.8 ± 0.2	8.1 ± 0.2	24.1 ± 0.8
pBI121	13	24.9 ± 0.2	28.5 ± 0.3	7.9 ± 0.2	24.7 ± 0.4
OE-1#13	10	32.3 ± 0.5*	42.2 ± 1.1*	8.7 ± 0.2*	35.7 ± 1.2*
OE-1#22	8	28.8 ± 0.4*	33.3 ± 0.4*	8.1 ± 0.3	31.9 ± 1*
OE-1#25	9	29.1 ± 0.6*	33.9 ± 0.5*	8.4 ± 0.3	30.1 ± 0.5*
OE-1#29	9	28.7 ± 0.4*	33.4 ± 0.6*	8.9 ± 0.3*	29.6 ± 0.6*
OE-2#24	12	31.6 ± 0.5*	37.2 ± 0.7*	8.6 ± 0.3	36.1 ± 1.2*
OE-2#45	10	32.5 ± 0.3*	38.8 ± 1.2*	8.8 ± 0.3*	38.2 ± 1.5*
OE-2#55	12	29.7 ± 0.3*	34.6 ± 0.5*	8.5 ± 0.3	31.8 ± 1.0*

The analysis was performed using four MiTFL1-1-overexpressing and three MiTFL1-2-overexpressing independent transgenic lines. The bolting time and rosette leaves were measured when the bolting height was 0.5–1 cm. The flowering time was considered the time when the first flowers opened. The plant height was measured 15 days after flowering. The error bars represent ±SD. The asterisks indicate significant differences (Duncan’s test: *P < 0.05)

**Fig. 6:**
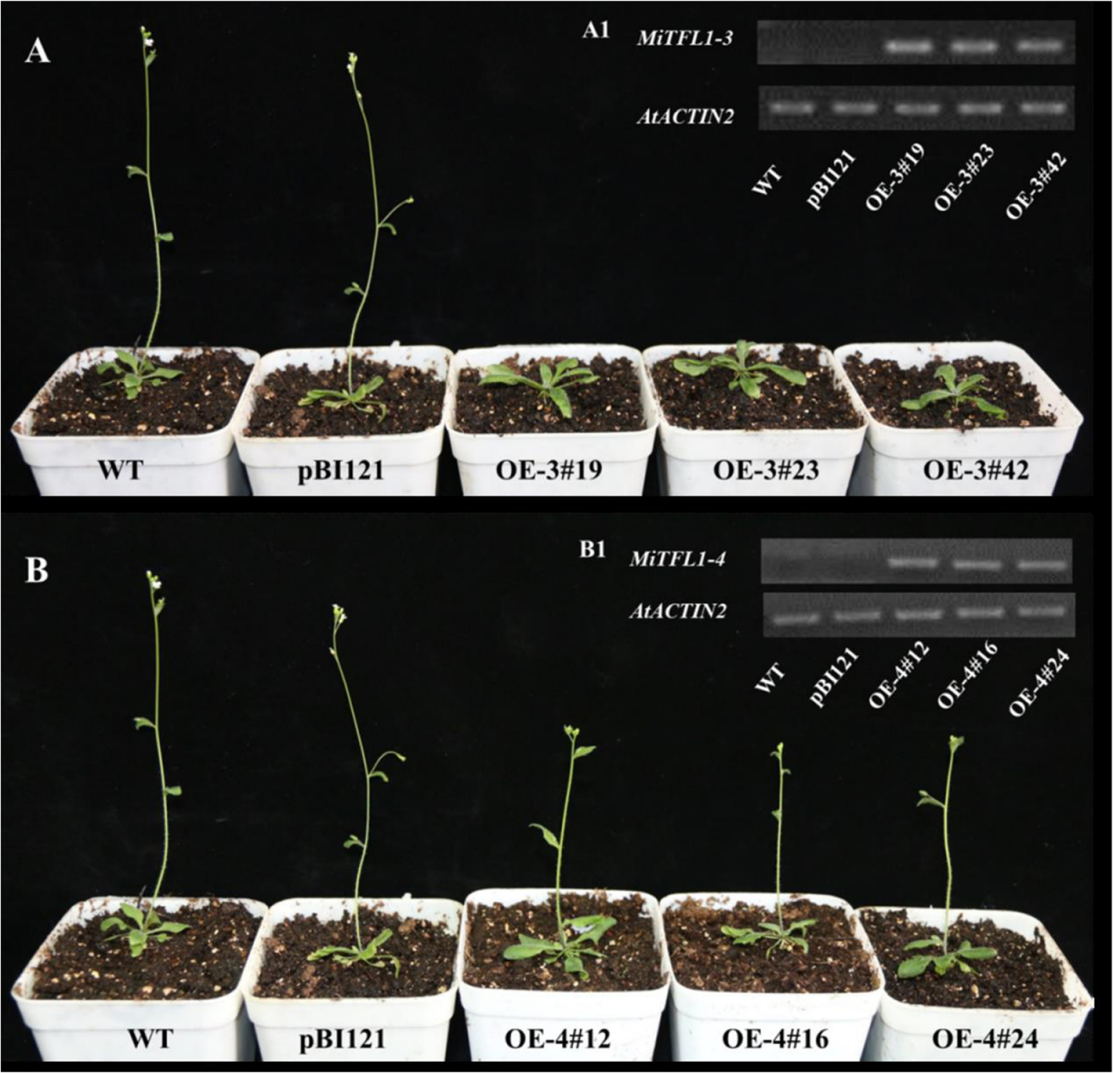
Phenotype of transgenic *Arabidopsis* lines and expression profiles of the transgenes. **A** Phenotype of MiTFL1-3-overexpressing transgenic lines showing delayed flowering (right) and that of WT and pBI121 transgenic lines as controls (left) under LD conditions. **A1** RT-PCR analysis of *MiTFL1-3* in the control and MiTFL1-3-overexpressing transgenic lines. **B** Phenotype of the MiTFL1-4-overexpressing transgenic lines, showing delayed flowering (right), and that of the WT and pBI121 transgenic lines as controls (left) under LD conditions. **B1** RT-PCR analysis of *MiTFL1-4* in the control and MiTFL1-4-overexpressing transgenic lines. The original data can be viewed in Fig. S1c-d

Three independent *MiTFL1-3* overexpression (OE-3#19, OE-3#23 and OE-3#42) and *MiTFL1-4* overexpression (OE-4#24, OE-4#45 and OE-4#55) lines were selected for functional analysis. Semiquantitative RT-PCR analysis demonstrated that *MiTFL1-3* and *MiTFL1-4* were abundantly expressed in the transgenic lines but absent in WT and pBI121 transgenic *Arabidopsis* plants (Fig. 6A1 and B1). The bolting time of the MiTFL1-3-overexpressing and MiTFL1-4-overexpressing transgenic plants was significantly delayed compared with that of the WT and pBI121 lines under long-day (LD) conditions (Fig. 6a and b and Table 2). The inhibitory effect of *MiTFL1-4* on flowering was weaker than those of the other three *MiTFL1* genes. The plant heights of some *MiTFL1-3* and *MiTFL1-4* transgenic lines showed significant differences, but the heights of some of the plants did not significantly differ from those of the control lines. The rosette leaves were not significantly affected in any of the plants (Table 2).

**Table 2:** Flowering phenotype analysis of WT, pBI121, MiTFL1-3-overexpressing (OE-3) and MiTFL1-4-overexpressing (OE-4) plants

ID	Number	Days to bolting (d)	Days to flowering (d)	No. of rosette leaves	Plant height (cm)
WT	10	24.8 ± 0.3	28.1 ± 0.3	8.1 ± 0.2	24 ± 2.9
pBI121	10	24.7 ± 0.3	27.4 ± 0.2	8.2 ± 0.4	25.3 ± 0.8
OE-3#19	12	37.0 ± 0.5*	47.3 ± 0.6*	8.5 ± 0.2	38.9 ± 1.1*
OE-3#23	11	30.9 ± 0.6*	38.9 ± 1.9*	8.4 ± 0.3	36.1 ± 1.2*
OE-3#42	12	30.2 ± 0.6*	34.7 ± 0.7*	8.3 ± 0.2	26.3 ± 2.0
OE-4#12	10	28.0 ± 0.7*	31.8 ± 0.6*	7.6 ± 0.3	26.9 ± 0.7
OE-4#16	10	26.8 ± 0.3*	30.7 ± 0.3*	8.2 ± 0.2	26.5 ± 0.3
OE-4#24	10	29.4 ± 0.8*	32.9 ± 0.8*	7.7 ± 0.3	31.2 ± 1.0*

The analysis was performed using three MiTFL1-3-overexpressing and three MiTFL1-4-overexpressing independent transgenic lines. The bolting time and rosette leaves were measured when the bolting height was 0.5–1 cm. The flowering time was considered the time when the first flowers opened. The plant height was measured 15 days after flowering. The error bars represent ±SD. The asterisks indicate significant differences (Duncan’s test: *P < 0.05)

#### *MiTFL1-1* and *MiTFL1-3* affect the flower phenotype in *Arabidopsis*

**Fig. 7:**
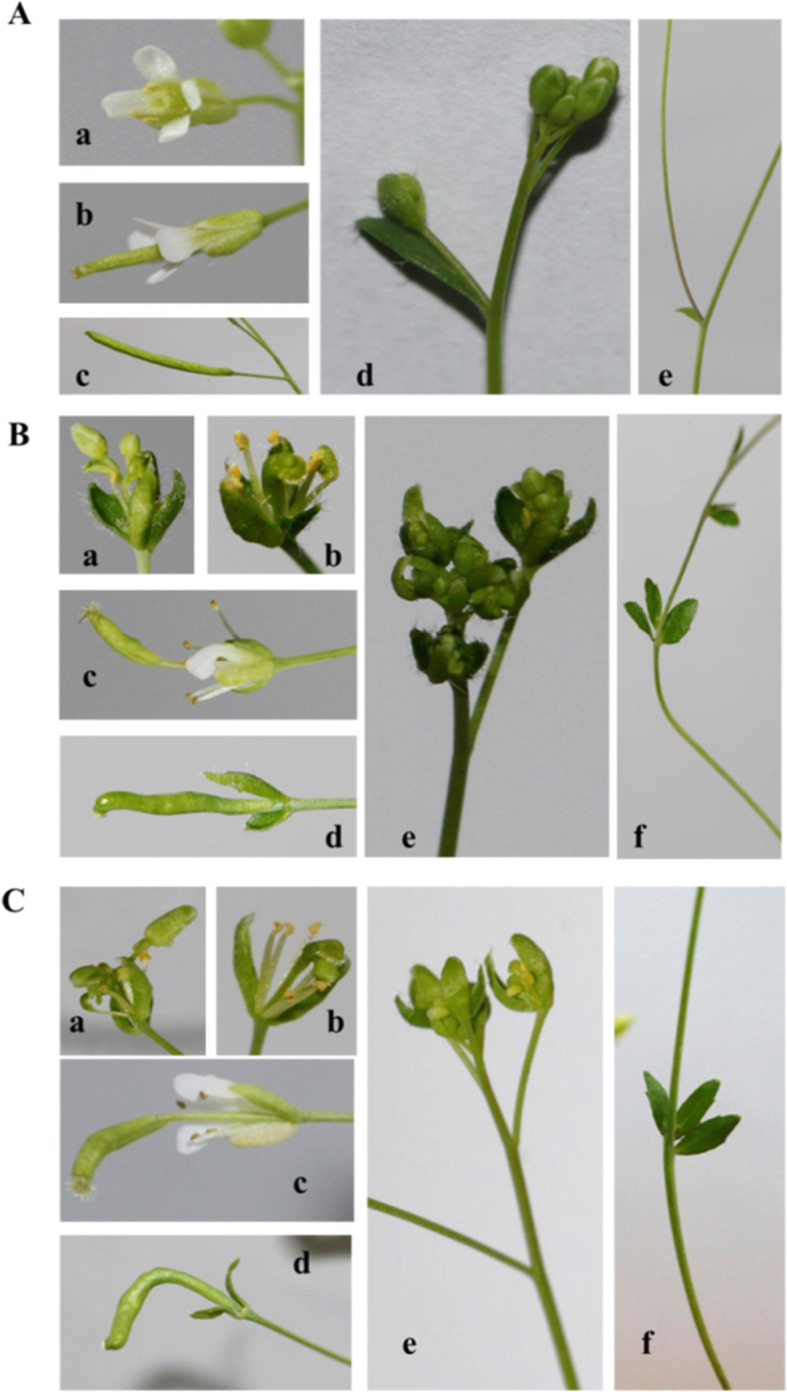
Photographs of the plant architecture, inflorescence, and floral phenotypes of WT and MiTFL1-1 and MiTFL1-3 transgenic *Arabidopsis* lines. **A** Phenotypes of WT *Arabidopsis*: (a, b) flowers, (c) silique, (d) inflorescence, and (f) stem. **B** Phenotypes of the MiTFL1-1-overexpressing line OE-1#13: (a) altered flower organs and no petals or carpels becoming a new inflorescence; (b) flower lacking petals; (c) a longer pod stalk formed in flowers; (d) pods with curved growth; (e) abnormal inflorescence; and (f) whorled leaves growing on the stem. **C** Phenotypes of the MiTFL1-3-overexpressing line OE-3#19: (a) altered flower organs and no petals or carpels becoming a new inflorescence; (b) flower lacking petals; (c) a longer pod stalk formed in flowers; (d) pods with curved growth; (e) abnormal inflorescence; and (f) whorled leaves growing on the stem

The MiTFL1-1-overexpressing (Fig. 7b) and MiTFL1-3-overexpressing transgenic lines (Fig. 7c) exhibited similar abnormal phenotypes to those of the WT lines (Fig. 7a). In the transgenic plants, some carpels developed into new inflorescences (Fig. 7B-a and C-a), and some flower structures lacked petals (Fig. 7B-b and C-b), in contrast with the results obtained for the WT plants (Fig. 7A-a and A-b). Two types of silique variations were found in the transgenic plants compared with the WT plants (Fig. 7A-b, A-c): in some siliques, the fruit stalk continued to lengthen from the flower during formation (Fig. 7B-c and C-c); in other siliques, the fruit stalk exhibited curved growth, and the siliques were shorter (Fig. 7B-c and C-c) than those of the WT plants. Additionally, the inflorescences of the transgenic plants were significantly different from those of the WT plants because of the variations in flower morphology (Fig. 7A-d, B-e and C-e). Furthermore, whorled leaves grew on the lateral branches of transgenic *Arabidopsis thaliana* but not in the control plants (Fig. 7A-e, B-f and C-f).

#### Expression patterns of endogenous genes in transgenic *Arabidopsis* expressing *MiTFL1* genes

**Fig. 8:**
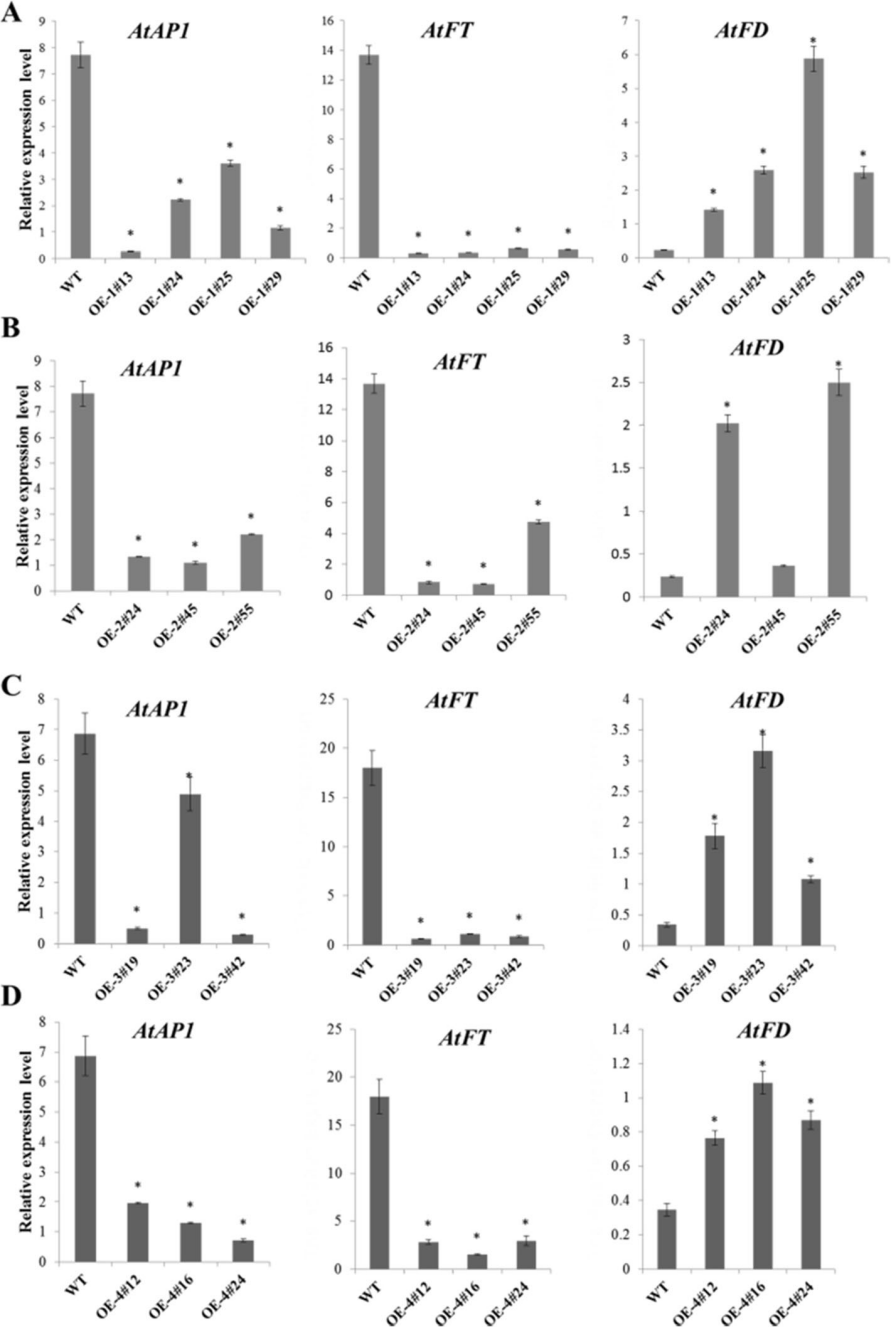
Expression analyses of flowering-related genes in transgenic *Arabidopsis*. **a-d** qRT-PCR analysis of endogenous flowering-related genes, including *Arabidopsis AtAP1*, *AtFT* and *AtFD*, in the MiTFL1-overexpressing and WT lines. The expression level was normalized to that of *Arabidopsis AtACTIN2*. The data are shown as the means ± SEs from three biological replicates. The significance of the differences among the samples was determined by Duncan’s test (*P* < 0.05)

To determine whether *MiTFL1* gene overexpression in transgenic *Arabidopsis* changed the expression of some flowering-related genes, such as the *AtFT*, *AtFD*, and *AtAP1* homologs in *Arabidopsis*, the entire aboveground portion of T3 generation homozygous transgenic *A. thaliana* was collected 30 days after planting and subjected to qRT-PCR analysis (Fig. 8). *AtACTIN2* was used as the internal reference gene. A similar expression pattern was found for the *AtFT*, *AtFD*, and *AtAP1* transcripts in *Arabidopsis* after overexpressing each of the four *MiTFL1* genes (Fig. 8a-d). The expression levels of the *AtFT* and *AtAP1* genes were significantly lower in all the *MiTFL1*-overexpressing transgenic lines than in the WT plants*.* However, the expression of the *AtFD* gene was significantly increased in many transgenic lines but not in the MiTFL1-2-overexpressing line OE-2#45.

### Proteins that interact with MiTFL1 proteins

**Fig. 9:**
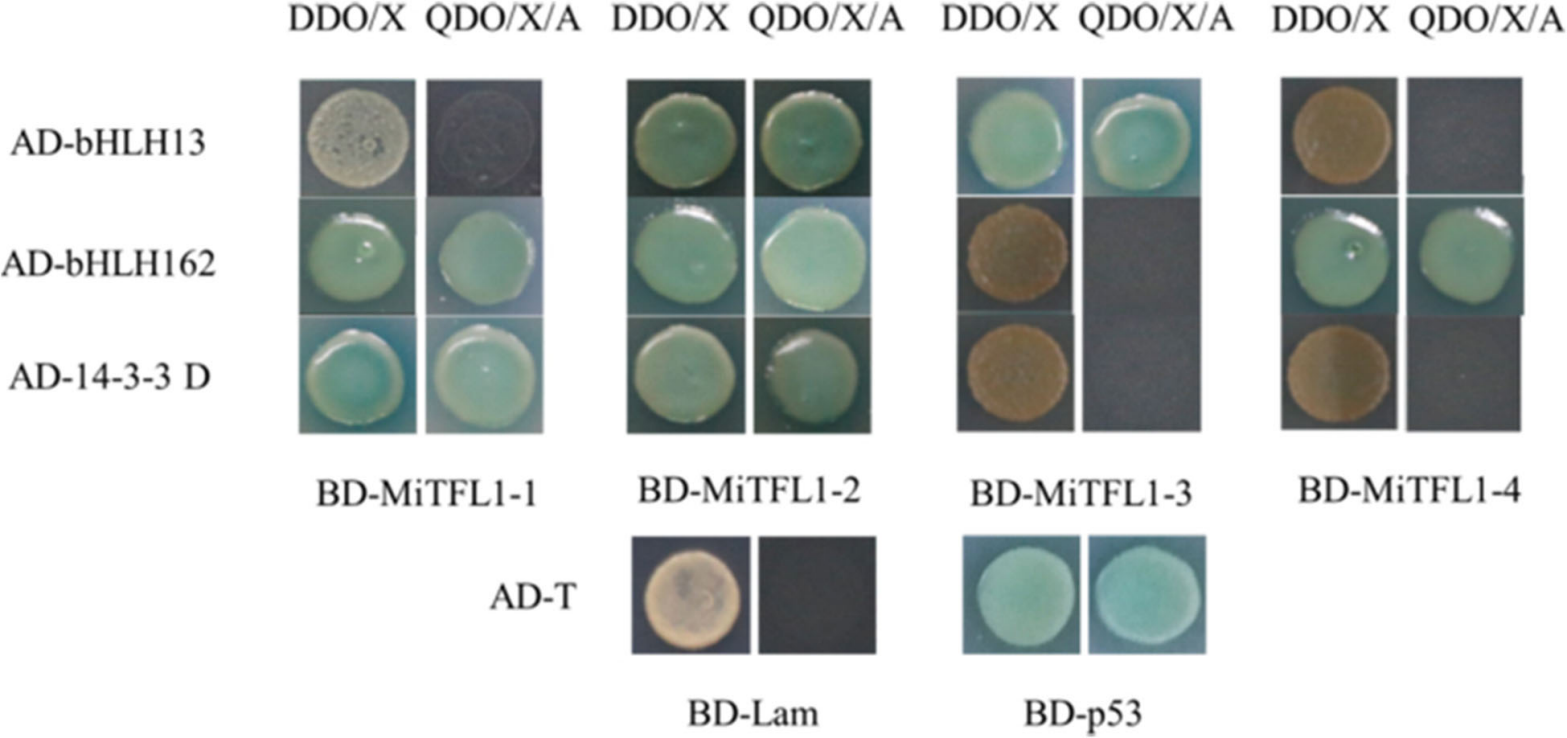
Identification of interactions between MiTFL1 proteins and other proteins in yeast. The interacting proteins were obtained through library screening; pGBKT7-53 and pGADT7-T served as positive controls, and pGBKT7-Lam and pGADT7-T were used as negative controls. The strains were cultured on DDO/−Trp/−Leu/X-α-gal (200 ng/ml) and QDO/−Trp/−Leu/−His/−Ade/X-α-gal (200 ng/ml)/AbA (500 μg/ml) media

The Y2H system was used to screen the proteins interacting with MiTFL1 proteins and verify their interactions. The bait vector pGBKT7-MiTFL1 was constructed by double enzyme digestion, and no autoactivation or toxicity was detected (shown in Fig. S2). Yeast cells with bait plasmids were combined with the cDNA homogenization library of ‘SiJiMi’ to screen for positive clones. After Y2H assays, we obtained 9 candidate proteins associated with the MiTFL1-1 protein, 7 with MiTFL1-2, 6 with MiTFL1-3, and 7 with MiTFL1-4 (Table S1). Three stress- or flowering-related proteins were selected from the candidate proteins for further point-to-point verification on DDO/X and QDO/X/A media. The three proteins were basic helix-loop-helix protein 13 (bHLH13), bHLH162 and 14-3-3D, as shown in Fig. 9. The cells with the candidate protein bHLH13 in the pGADT7 recombinant vector turned blue and exhibited normal growth on QDO/X/A solid medium, indicating that the protein interacts with MiTFL1-2 and MiTFL1-3 proteins. bHLH162 interacted with MiTFL1-1, MiTFL1-2 and MiTFL1-4, whereas 14-3-3D interacted with only MiTFL1-1 and MiTFL1-2.

## Discussion

*FT* and *TFL1* encode a pair of flowering regulators belonging to the PEBP family and play critical roles during the switch from vegetative growth to reproductive development [19]. *FT* and *TFL1* share high homology, as demonstrated by their high nucleotide and amino acid sequence identities, but have opposite functions. *FT* promotes the transition to reproductive development and flowering, whereas *TFL1* represses flowering [19, 29]. Our previous study identified three *FT* genes from mango and further confirmed that all *FT* genes significantly promoted flowering in the transgenic plants [27]. In the present study, four *TFL1* genes were obtained from transcriptomic and genomic data analysis of ‘SiJiMi’ mango (unpublished data) and were named *MiTFL1-1*, *MiTFL1-2, MiTFL1-3* and *MiTFL1-4*. Sequence analysis showed that the four *TFL1* genes of ‘SiJiMi’ mango were highly consistent with the nucleotide sequences of ‘Alphonso’ (Fig. S3).

Multiple copies of *TFL1* homologous genes are also found in other plants, such as two in soybean [30], moso bamboo [13] and loquat [31], three in petunia [32], four in cotton [33] and five in *Hevea brasiliensis* [34]. In a previous study, only two *TFL1* genes, namely, *MiTFL1* and *MiTFL1a,* were found in another mango variety, ‘*Alphonso*’, corresponding to *MiTFL1-2* and *MiTFL1-1*, respectively, in the present study [35]. *MiTFL1* genes contain four exons and three introns. The nucleotide lengths of the second and third exons were highly consistent among different *MiTFL1* genes (Fig. 1a). The critical amino acids His88 and Asp144 of TFL1 proteins were also found in these four MiTFL1 proteins (Fig. 1b). The constructed phylogenetic tree indicated that the four MiTFL1 proteins belong to the TFL1 protein branch. In the tree, MiTFL1-1 and MiTFL1-2 were close to each other, whereas MiTFL1-3 and MiTFL1-4 were also located close to each other (Fig. 2).

The *TFL1* gene expression pattern is related to floral development in most fruit trees*.* Citrus *CsTFL1* is expressed only in floral organs [36]. *P. mume PmTFL1* is expressed in the leaves, stems, and roots in the juvenile phase, whereas *PmTFL1* has been detected only in leaf buds and young leaves at the mature stage [16]. The *PpTFL1* gene of peach is mainly expressed in mature young leaves but is not found in mature leaves and flower organs [37]. *EjTFL1-1* is mainly expressed in roots and leaf buds but is expressed at low levels in shoots, flower buds and flowers. *EjTFL1-2* is mainly expressed in leaf buds, flowers, and fruits and is not expressed in other tissues [31]. In the present study, *MiTFL1* genes showed different expression patterns compared to those reported above. Tissue expression analysis showed that *MiTFL1-1*, *MiTFL1-2*, *MiTFL1-3* and *MiTFL1-4* were expressed in both vegetative and reproductive tissues but differed in expression levels. Our results were similar to those found in other mango varieties, namely, ‘Alphonso’ and ‘Ratna’ [35]. These results indicated that the tissue expression pattern of the *TFL1* gene was significantly different among different species.

Some studies have shown that the expression pattern of the *TFL1* gene is different at different stages of floral development. For example, in pear, apple, and quince, the *TFL1-1* and *TFL1-2* genes are highly expressed in buds before floral differentiation, and their expression appears to decrease after floral differentiation [38]. In *H. brasiliensis*, *HbTFL1-1*, *HbTFL1-2* and *HbTFL1-3* expression increases gradually during inflorescence development, but the expression of *HbCEN1* and *HbCEN2* continuously decreases over this period [39]. The expression levels of *EjTFL1-1* and *EjTFL1-2* gradually decrease before floral bud differentiation and start to increase again during the flower-opening period [31]. In the mango variety ‘*Alphonso*’, *MiTFL1* expression increases during the flowering induction period and subsequently decreases, whereas *MiTFL1a* expression remains low during the flowering period [35]. In this study, the expression of the *MiTFL1* gene in mango was similar to that reported above. *MiTFL1-1* and *MiTFL1-2* were highly expressed in leaves at the floral induction stage, while *MiTFL1-3* and *MiTFL1-4* were highly expressed in leaves at the floral differentiation stage.

*TFL1* homologous genes have similar functions in many species, and these functions include delaying the flowering time and maintaining the inflorescence meristem through suppression of *AP1* and *LFY* expression [15]. Overexpression of apple *MdTFL1* in *Arabidopsis* delays flowering time, and inhibition of the expression of the *MdTFL1* gene by RNAi technology results in early flowering traits [34, 40]. The Japanese apricot *PmTFL1* [16] and five rubber *TFL1* genes showed the same function in transgenic *Arabidopsis* [39]. In the present study, four mango *MiTFL1* genes had the same function of delaying flowering time in transgenic *Arabidopsis*. Moreover, the *MiTFL1-1* and *MiTFL1-3* transgenic lines exhibited abnormal flower organ phenotypes, such as missing petals, carpel development into a new inflorescence, curved pod growth and seed abortion. These results suggest that *MiTFL1-1* and *MiTFL1-3* are involved in flower organ development. Similar phenotypes were also found in chrysanthemum *CmTFL1c-* and *Prunus PsTFL1*-overexpressing transgenic *Arabidopsis* lines [41, 42].

Introduction of the exogenous *TFL1* gene significantly downregulated the expression levels of the endogenous genes *AtFT* and *AtAP1* in transgenic *Arabidopsis*, leading to delayed flowering. For example, overexpressing the *HkTFL1* gene in *Hemerocallis* delayed flowering, and the expression levels of *AtFT* and *AtAP1* in transgenic *Arabidopsis* were decreased compared with those in WT *Arabidopsis* [43]. The *Chrysanthemum morifolium CmTFL1c* gene negatively regulates flowering by inhibiting *AtFT*, *AtLFY* and *AtAP1* expression [42]. Cucumber *CsTFL1b* also delays flowering in *Arabidopsis* and decreases and increases the expression levels of *AtFT* and *AtFD*, respectively [4]. In the present study, we found that four *MiTFL1* genes downregulated *AtFT* and *AtAP1* expression but upregulated *AtFD* expression in transgenic *Arabidopsis*.

In rice, the TFL1-like protein RICE CENTRORADIALIS (RCN) can directly interact with the 14-3-3 protein [44]. In this study, we also screened some proteins interacting with MiTFL1 using Y2H assays (Table S1). For example, the 14-3-3D protein was found to interact with MiTFL1-1 and MiTFL1-2, and this result was similar to that found in rice. Two other bHLH proteins were also found to interact with different MiTFL1 proteins and play a role in several processes, including growth, development, and the response to various stresses [45]. Additionally, other proteins that may interact with MiTFL1 proteins have been screened, and they are also involved in the stress response, plant growth and development. However, these interacting proteins must be further tested.

## Conclusions

In the present study, four *MiTFL1* genes were identified in mango. These proteins contained both the critical amino acids His88 and Asp144. Expression analysis showed that the *MiTFL1* genes exhibited a similar expression pattern: the *MiTFL1* genes were expressed in vegetative and reproductive tissues and were highly expressed in mature leaves during the flowering induction period and floral differentiation stage. Overexpression of the four *MiTFL1* genes in *Arabidopsis* resulted in delayed flowering, whereas *MiTFL1-1* and *MiTFL1-3* overexpression affected floral organ development. Y2H analysis showed that the MiTFL1 proteins interact with bHLH and 14-3-3 proteins. These results provide preliminary evidence that *MiTFL1* genes negatively regulate floral induction in mango, but their interaction mechanisms must be further validated.

## Methods

### Plant materials and growth conditions

The *M. indica* L*.* cv*.* ‘SiJiMi’ plants used in this study were planted in an orchard at Guangxi University, Nanning, Guangxi, China (22°502 N, 108°17′E). For tissue expression analysis, leaves, stems and flowers were collected from 17-year-old trees on 10 March 2021. For seasonal expression analysis, leaves (closer to the terminal bud) were collected once per month from 1 November 2020 (vegetative period) to 1 May 2021 (fruit development period). All the samples were immediately frozen in liquid nitrogen and stored at − 80 °C. The *Arabidopsis* ecotype Col-0 was maintained in our laboratory.

### Isolation of *MiTFL1* genes from mango

Total RNA was extracted from mango leaves using the RNAprep Pure Plant Kit (TianGen, Beijing, China) according to the manufacturer’s instructions. First-strand cDNA was synthesized from 1 μg of total RNA using M-MLV reverse transcriptase (TaKaRa, Dalian, China) according to the manufacturer’s instructions. Genomic DNA was extracted from mango leaves using the CTAB method. Four *TFL1* genes were obtained from mango leaves and named *MiTFL1-1*, *MiTFL1-2*, *MiTFL1-3* and *MiTFL1-4*. Specific primers (QTFL1-1u/d, QTFL1-2u/d, QTFL1-3u/d, and QTFL1-4u/d; Table S2) were designed to amplify *MiTFL1* genes from genomic DNA and cDNA. The polymerase chain reaction (PCR) mixture contained 2.5 μl of 10× PCR buffer (with MgCl^2+^), 0.5 μl of 10 mM dNTPs (Sangon Biotech, Shanghai, China), 1 μl of each of the upstream and downstream primers (10 μM), 0.15 μl of TransTaq-T DNA polymerase (TianGen), 1 μl of genomic DNA (100 ng/μl) or cDNA (100 ng/μl), which served as the templates, and sterile water (25 μl). The PCR amplification conditions included an initial denaturation step of 4 min at 95 °C; 38 cycles of 95 °C for 40 s, 56 °C for 50 s, and 72 °C for N min (*N* = 1 min/kb); and a final extension at 72 °C for 10 min. The amplified fragments were cloned into the pMD18-T vector (Takara) and then sequenced.

### Sequence analysis

Sequence analysis and amino acid prediction were performed using BioXM 2.6 software. IBS version 1.0 was used to generate exon-intron structures. The conserved domains were analyzed using the NCBI BLAST search engine (https://www.ncbi.nlm.nih.gov/Structure/cdd/wrpsb.cgi). The amino acid sequences of the FT1/TFL1 family were downloaded through BLAST searches of GenBank (http://www.ncbi.nlm.nih.gov/BLAST/). Multiple sequence alignments of TFL1 proteins were analyzed using DNAMAN software. The phylogenetic tree was constructed using the neighbor-joining method in MEGA-Χ with 1000 bootstrap replicates.

### Expression analysis of *MiTFL1* genes

The expression of *MiTFL1* genes was detected by quantitative real-time PCR. Total RNA from all the samples was extracted using the RNAprep Pure Plant Kit (TianGen) according to the manufacturer’s instructions. First-strand cDNA was synthesized and used as a template. Gene-specific primers (qTFL1-1u/d, qTFL1-2u/d, qTFL1-3u/d and qTFL1-4u/d; Fig. S4) were designed to distinguish the *MiTFL1* genes. The *MiActin1* gene of mango was used as the internal reference gene [46]. The PCR mixture contained 10 μl of SYBR Premix Ex Taq II (Takara), 1 μl of cDNA (100 ng/μl), 0.5 μl (10 μM) of the upstream and downstream primers, 0.8 μl of ROX Reference Dye II, and sterile water to obtain a total volume of 20 μl. The PCR amplification conditions included 30 s at 95 °C; 40 cycles of 95 °C for 5 s, 60 °C for 34 s, and 95 °C for 15 s; 60 °C for 1 min; and 95 °C for 15 s. The relative transcript abundances were estimated using the 2^-ΔΔCt^ method [47]. The analysis of each sample included three biological replicates.

### Subcellular localization of MiTFL1 proteins

The complete coding sequences of *MiTFL1* genes without terminator codons were constructed into the P1300-GFP vector between the *Xba*I and *BamH*I restriction enzyme cleavage sites. The constructed vectors were transformed into *Agrobacterium tumefaciens* EHA105. The GFP fusion vectors and empty vector were subsequently transformed into onion epidermal cells via *A. tumefaciens* EHA105. 4′,6-Diamidino-2-phenylindole (DAPI) was used to visualize the nucleus. Images were captured using a high-resolution laser confocal microscope (TCS-SP8MP; Leica, Germany).

### Plasmid construction and genetic transformation

The *MiTFL1*-overexpressing (OE) vectors were constructed by cloning the genes into the pBI121 binary vector using CaMV 35S as the promoter between the *Xba*I and *Xma*I restriction enzyme cleavage sites. The overexpression plasmids were transferred into the *A. tumefaciens* strain EHA105. The overexpression vectors and empty vector were subsequently transformed into WT *A. thaliana* using the floral-dip method [48]. The transgenic seeds were selected on 1/2 MS medium containing 50 mg/l kanamycin and confirmed by genomic PCR. The specific primers MiTFL1-1u/d, MiTFL1-2u/d, MiTFL1-3u/d and MiTFL1-4u/d were used to detect whether transformation of the *MiTFL1* genes was successful. Homozygous T3 transgenic plants were used for subsequent experiments.

### Phenotypic analyses

Wild-type and empty vector-transformed *Arabidopsis* plants were used as controls. Several phenotypic indexes, including the bolting time, flowering time, time from bolting to flowering, and rosette leaves, were measured. To detect the expression levels of the *MiTFL1* genes and some flowering-related genes in transgenic and control plants, 30-day-old seedlings of both transgenic and WT *Arabidopsis* plants were collected for total RNA extraction. Total RNA was extracted, and first-strand cDNA was synthesized as described above. Semiquantitative RT-PCR was performed to determine the expression levels of the *MiTFL1* genes in the transgenic and control lines. The PCR amplification conditions comprised an initial denaturation step of 2 min at 95 °C; 30 cycles of 95 °C for 30 s, 56 °C for 30 s, and 72 °C for 30 s; and a final extension at 72 °C for 5 min. The PCR products were electrophoretically separated on a 1.8% agarose gel. The expression levels of some endogenous flowering-related genes in transgenic and control *Arabidopsis* lines were detected by qRT-PCR using the reaction system and conditions described above. *Arabidopsis AtACTIN2* was used as the internal reference gene for qRT-PCR analysis. All the primers used in this study are listed in Table S1.

### Proteins interacting with the MiTFL1 proteins

A cDNA library from *M. indica* L*.* cv*.* SiJiMi leaves and flowering organs was constructed using the Yeast Two-Hybrid Library Construction Kit (Clontech, Dalian, China). Y2H assays were performed according to the Yeastmaker™ Yeast Transformation System 2 protocol (Clontech). The complete coding sequences of the *MiTFL1* genes were inserted into the pGBKT7 vector between the *Nde*I and *EcoR*I restriction enzyme cleavage sites. The pGBKT7-bait plasmid was transformed into competent Y2H Gold yeast cells, which were diluted 10^_1^ and 10^_2^ and coated on SDO/−Trp, SDO/−Trp/X-alpha-Gal and SDO/−Trp/X-Alpha-Gal/AbA media. The transcriptional activity and toxicity were verified using this method.

The interacting proteins were identified by screening a DNA library on QDO/−Trp/−Leu/−His/−Ade culture medium. The plasmids of the interacting proteins were extracted and used for further verification of the actual interactions. Y2H Gold yeast cells containing the pGBKT7-bait plasmid and Y187 yeast cells containing the candidate prey were mixed and cultured in liquid medium containing 2 × YPDA at 30 °C and 200 rpm for 20–24 h. The mixture was then coated on DDO/−Trp/−Leu/X and QDO/X/A media and cultured for 3–5 days. Blue colonies on the media indicated a positive interaction. The AbA concentration was 500 μg/ml, and the X-α-gal concentration was 200 ng/ml. Y2H Gold (pGBKT7-53) and Y187 (pGADT7-T) served as positive controls, and Y2H Gold (pGBKT7-LAM) and Y187 (pGADT7-T) were used as negative controls.

### Statistical analysis

SPSS 19.0 statistical software (SPSS Inc., Chicago, IL, United States) was used for the statistical analyses.

## Supplementary Information

**Additional file 1 **: **Supplementary Table 1.** Four TFL1 interacting protein through yeast two hybrid.

**Additional file 2 **: **Supplementary Table 2**. Primers used in this study.

**Additional file 3 **: **Supplement Figure 1**. Source data for Figs. 5 and 6. (a) The red frame indicates the source data in Fig. S1a displayed for Fig. 5a-a1. (b) The source data in Fig. S1b displayed for Fig. 5b-b1. (c) The source data in Fig. S1c displayed for Fig. 6a-a1. (d) The source data in Fig. S1d displayed for Fig. 6b-b1.

**Additional file 4 **: **Supplement Figure 2**. The autoactivation and toxicity of pGBKT7-MiTFL1s vector, concentrations of 10^− 1^. Yeast bait expression vectors of MiTFL1-1, MiTFL1-2, MiTFL1-3 and MiTFL1-4 were constructed by double enzyme digestion method, and transferred into Y2H Gold yeast, which were cultured in SDO/−Trp, SDO/X and SDO/X/A medium. Y2H Gold (pGBKT7-53) served as positive controls, and Y2H Gold (pGBKT7-lam) were used as negative controls. The results showed that the four pGBKT7-MiTFL1s had no autoactivation and toxicity.

**Additional file 5 **: **Supplement Figure 3**. Comparison of cDNA sequences of four *MiTFL1* genes in two cultivars of ‘Alphonso’ and ‘SiJiMi’. (A) The comparative similarity between the two cultivars of *MiTFL1-1* gene was 99.4%. (B) The comparative similarity between the two cultivars of *MiTFL1-2* gene was 100.0%. (C) The comparative similarity between the two cultivars of *MiTFL1-3* gene was 98.5%. (D) The comparative similarity between the two cultivars of *MiTFL1-4* gene was 98.3%.

**Additional file 6 **: **Supplement Figure 4**. The primer design site of *MiTFL1s* gene was used for qRT-PCR. cDNA sequence comparison of the four genes, where the yellow shaded part represents the start codon and the red shaded part represents the stop codon. The red sequence represents the upstream primer sequence and the blue sequence represents the downstream primer sequence. The black part indicates a similarity of 100%.

## Data Availability

All the data generated or analyzed during this study are included in this published article and its supplementary information files. The datasets generated in this study are available in GenBank (http://www.ncbi.nlm.nih.gov/Genbank), and the accession numbers are as follows: AtTFL1 (NP_196004.1), CsTFL1 (NP_001275848), DlTFL1 (AHY24028.1), JrTFL1 (XP_018811176.1), MdTFL1 (NP_001280887.1), PpTFL1 (NP_001289244.1), VvTFL1A (NP_001267929.1), MdCEN (NP_001280940.1), PvCEN protein 1 (XP_031269481.1), TcCEN (XP_017973069.1), HmTFL1 (MF374628.1), GmTFL1.3 (FJ573238.1), FcTFL1-2 (AB746842.1), CcTFL1 (XP_006430226.1), ClTFL1 (AWW25018.1), FcTFL1 (BAX00857.1), NsTFL1 (XP_009766168.1), PmTFL1 (AEO72021.1), PyTFL1 (AEO72023.1), VvTFL1C (NP_001267933.1), ZjTFL1 (XP_015898753.1), AcFT (AJA40932.1), AtFT (BAA77838.1), EdFT (AMB72867.1), MiFT (AGA19021.1), VvFT (NP_001267907.1), AtMFT (OAP13671.1), ClMFT (AWW25016.1), DlMFT (AUG98253.1), and VvMFT (NP_001267935.1).

## Abbreviations

CTAB: Hexadecyltrimethylammonium bromide
qRT-PCR: Quantitative reverse transcription-polymerase chain reaction
DAPI: 6-diamidino-2-phenylindole
X-Alpha-Gal: 5-bromo-4-chloro-3-indoxyl-α-D-galactopyranoside
AbA: Aureobasidin A
