# Supplementary material for: Overexpression of four MiTFL1 genes from mango delays the flowering time in transgenic Arabidopsis
Source: BMC Plant Biol. 2021 Sep 7;21:407. doi: 10.1186/s12870-021-03199-9 (PMC8422776; doi:10.1186/s12870-021-03199-9)

**The Overexpression of Four *MiTFL1* Genes from Mango Delays the Flowering Time in Transgenic *Arabidopsis***

Yi-Han Wang*, Xin-Hua He*, Hai-Xia Yu, Xiao Mo, Yan Fan, Zhi-Yi Fan, Xiao-Jie Xie, Yuan Liu, Cong Luo**

*College of Agriculture, State Key Laboratory for Conservation and Utilization of Subtropical Agro-Bioresources, Guangxi University, Guangxi Nanning, 530004*

**These authors contributed equally to this work.*

***Corresponding author: Cong Luo, 22003luocong@163.com*

Supplement Figure 1

Source data for Figs. 5 and 6. (a) The red frame indicates the source data in Fig. S1a displayed for Fig. 5a-a1. (b) The source data in Fig. S1b displayed for Fig. 5b-b1. (c) The source data in Fig. S1c displayed for Fig. 6a-a1. (d) The source data in Fig. S1d displayed for Figs. 6b-b1.


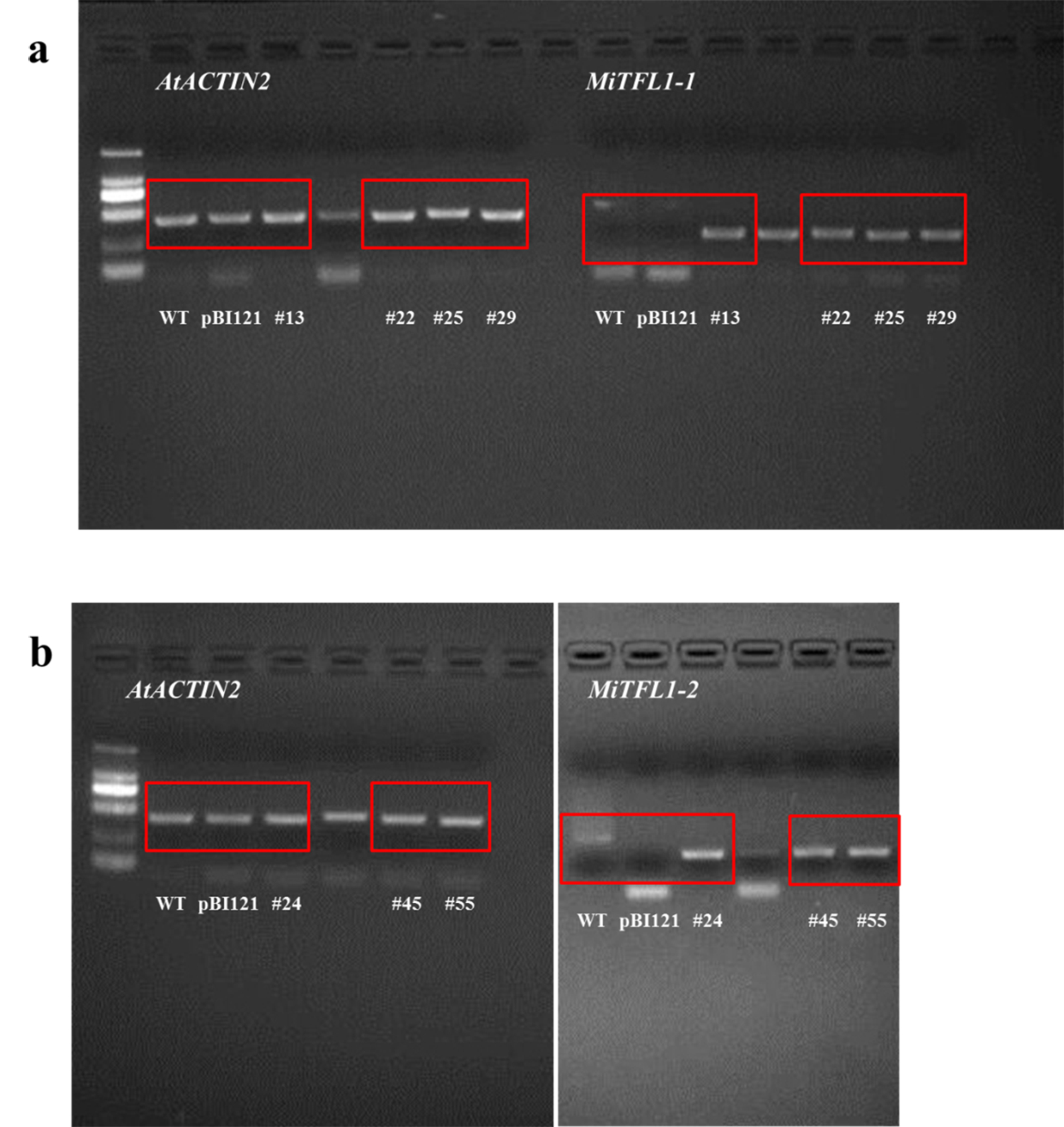


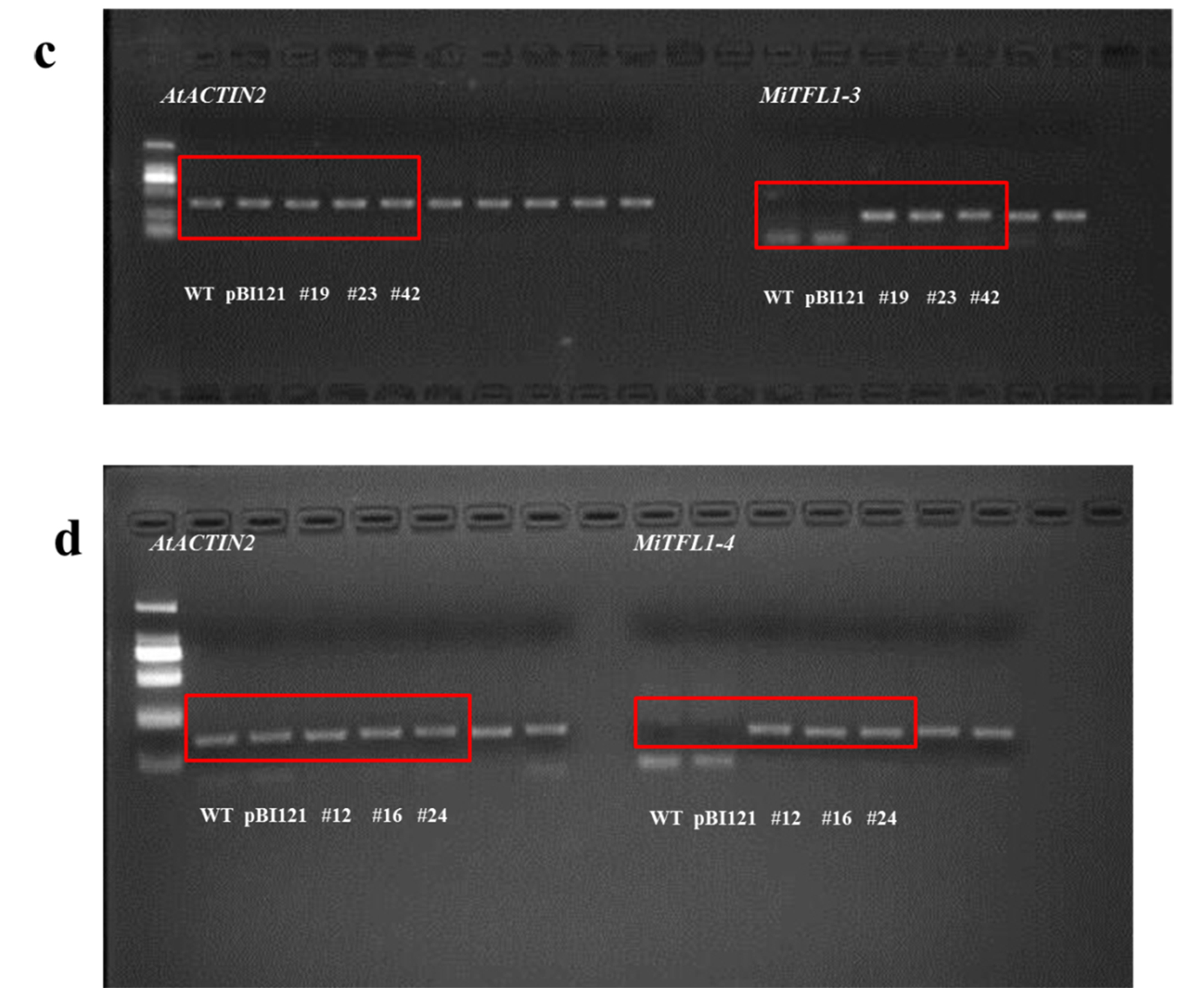

Supplement: Supplementary file 3 — Additional file 3 : Supplement Figure 1. Source data for Figs. 5 and 6. (a) The red frame indicates the source data in Fig. S1a displayed for Fig. 5a-a1. (b) The source data in Fig. S1b displayed for Fig. 5b-b1. (c) The source data in Fig. S1c displayed for Fig. 6a-a1. (d) The source data in Fig. S1d displayed for Fig. 6b-b1. [file 12870_2021_3199_MOESM3_ESM.docx]
