# Supplementary material for: Overexpression of four MiTFL1 genes from mango delays the flowering time in transgenic Arabidopsis
Source: BMC Plant Biol. 2021 Sep 7;21:407. doi: 10.1186/s12870-021-03199-9 (PMC8422776; doi:10.1186/s12870-021-03199-9)

**The Overexpression of Four *MiTFL1* Genes from Mango Delays the Flowering Time in Transgenic *Arabidopsis***

Yi-Han Wang*, Xin-Hua He*, Hai-Xia Yu, Xiao Mo, Yan Fan, Zhi-Yi Fan, Xiao-Jie Xie, Yuan Liu, Cong Luo**

*College of Agriculture, State Key Laboratory for Conservation and Utilization of Subtropical Agro-Bioresources, Guangxi University, Guangxi Nanning, 530004*

**These authors contributed equally to this work.*

***Corresponding author: Cong Luo, 22003luocong@163.com*

Supplement Figure 2

The autoactivation and toxicity of pGBKT7-MiTFL1s vector, concentrations of 10^-1^. Yeast bait expression vectors of MiTFL1-1, MiTFL1-2, MiTFL1-3 and MiTFL1-4 were constructed by double enzyme digestion method, and transferred into Y2H Gold yeast, which were cultured in SDO/-Trp, SDO/X and SDO/X/A medium. Y2H Gold (pGBKT7-53) served as positive controls, and Y2H Gold (pGBKT7-lam) were used as negative controls. The results showed that the four pGBKT7-MiTFL1s had no autoactivation and toxicity.


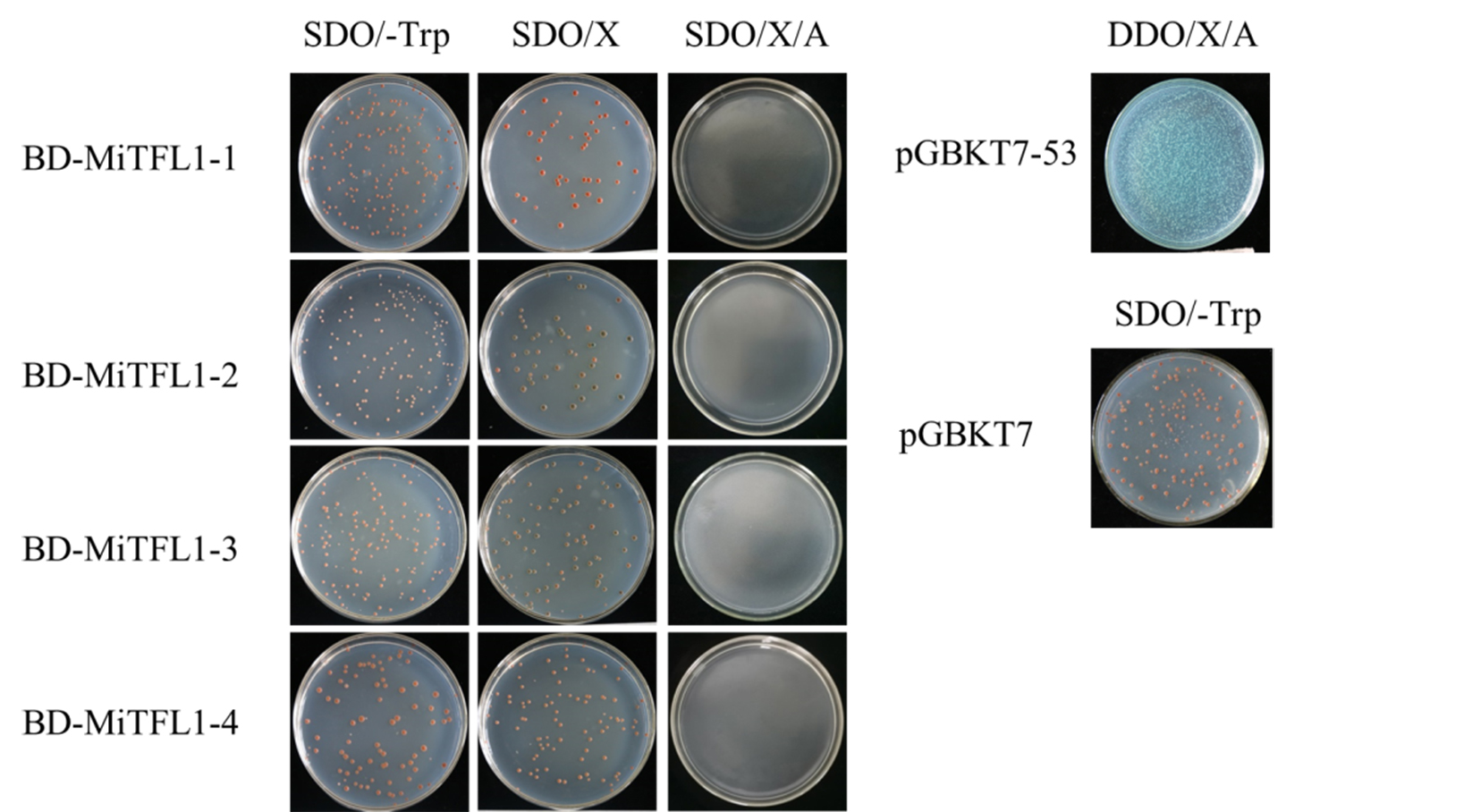

Supplement: Supplementary file 4 — Additional file 4 : Supplement Figure 2. The autoactivation and toxicity of pGBKT7-MiTFL1s vector, concentrations of 10− 1. Yeast bait expression vectors of MiTFL1-1, MiTFL1-2, MiTFL1-3 and MiTFL1-4 were constructed by double enzyme digestion method, and transferred into Y2H Gold yeast, which were cultured in SDO/−Trp, SDO/X and SDO/X/A medium. Y2H Gold (pGBKT7-53) served as positive controls, and Y2H Gold (pGBKT7-lam) were used as negative controls. The results showed that the four pGBKT7-MiTFL1s had no autoactivation and toxicity. [file 12870_2021_3199_MOESM4_ESM.docx]
