# Supplementary material for: Overexpression of four MiTFL1 genes from mango delays the flowering time in transgenic Arabidopsis
Source: BMC Plant Biol. 2021 Sep 7;21:407. doi: 10.1186/s12870-021-03199-9 (PMC8422776; doi:10.1186/s12870-021-03199-9)

**The Overexpression of Four *MiTFL1* Genes from Mango Delays the Flowering Time in Transgenic *Arabidopsis***

Yi-Han Wang*, Xin-Hua He*, Hai-Xia Yu, Xiao Mo, Yan Fan, Zhi-Yi Fan, Xiao-Jie Xie, Yuan Liu, Cong Luo**

*College of Agriculture, State Key Laboratory for Conservation and Utilization of Subtropical Agro-Bioresources, Guangxi University, Guangxi Nanning, 530004*

**These authors contributed equally to this work.*

***Corresponding author: Cong Luo,* [*22003luocong@163.com*](mailto:22003luocong@163.com)

Supplement Figure 3

Comparison of cDNA sequences of four *MiTFL1* genes in two cultivars of ‘Alphonso’ and ‘SiJiMi’. (A) The comparative similarity between the two cultivars of *MiTFL1-1* gene was 99.4%. (B) The comparative similarity between the two cultivars of *MiTFL1-2* gene was 100.0%. (C) The comparative similarity between the two cultivars of *MiTFL1-3* gene was 98.5%. (D) The comparative similarity between the two cultivars of *MiTFL1-4* gene was 98.3%.


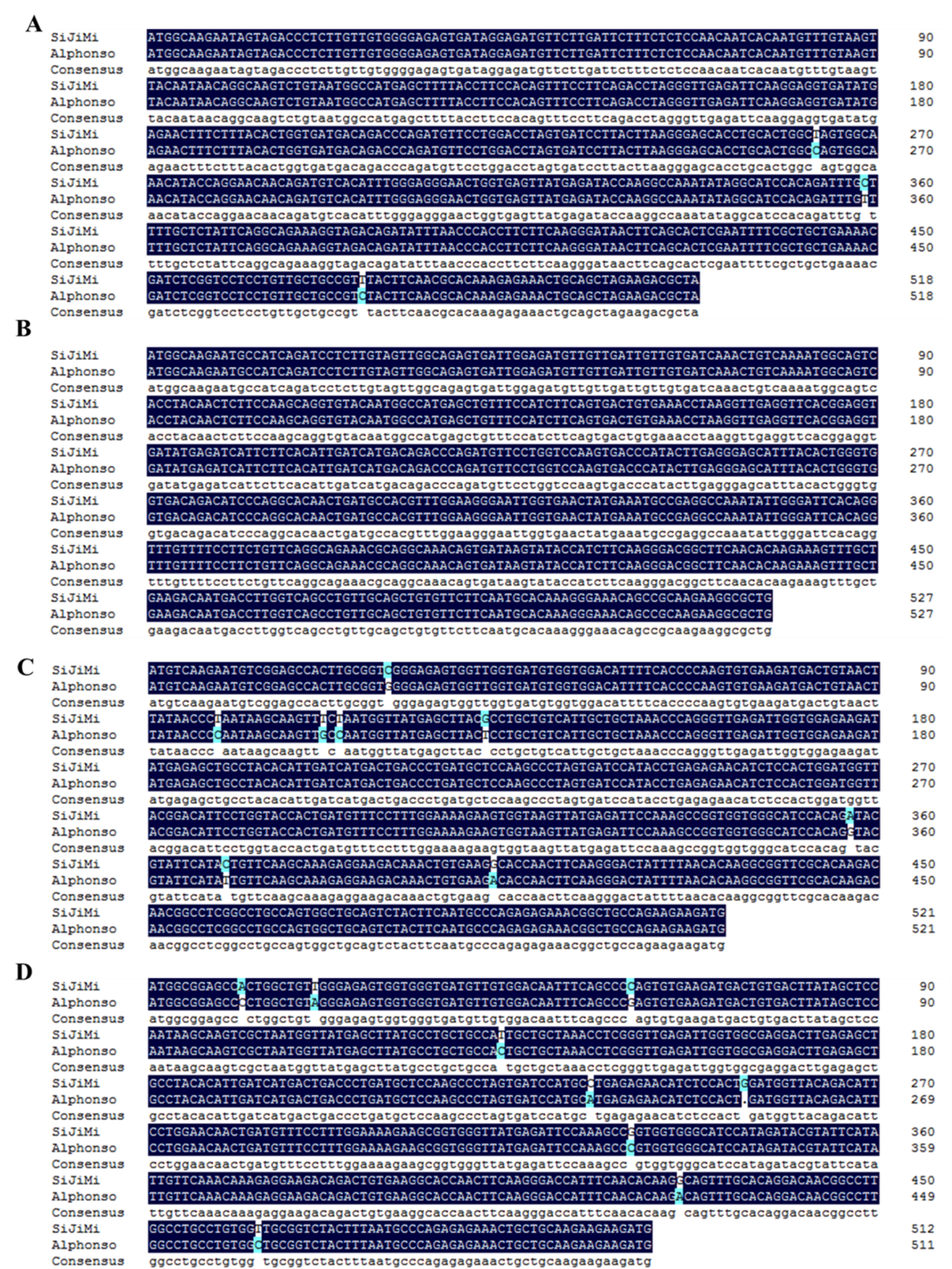

Supplement: Supplementary file 5 — Additional file 5 : Supplement Figure 3. Comparison of cDNA sequences of four MiTFL1 genes in two cultivars of ‘Alphonso’ and ‘SiJiMi’. (A) The comparative similarity between the two cultivars of MiTFL1-1 gene was 99.4%. (B) The comparative similarity between the two cultivars of MiTFL1-2 gene was 100.0%. (C) The comparative similarity between the two cultivars of MiTFL1-3 gene was 98.5%. (D) The comparative similarity between the two cultivars of MiTFL1-4 gene was 98.3%. [file 12870_2021_3199_MOESM5_ESM.docx]
