# Supplementary material for: Overexpression of four MiTFL1 genes from mango delays the flowering time in transgenic Arabidopsis
Source: BMC Plant Biol. 2021 Sep 7;21:407. doi: 10.1186/s12870-021-03199-9 (PMC8422776; doi:10.1186/s12870-021-03199-9)
Supplement: Supplementary file 6 — Additional file 6 : Supplement Figure 4. The primer design site of MiTFL1s gene was used for qRT-PCR. cDNA sequence comparison of the four genes, where the yellow shaded part represents the start codon and the red shaded part represents the stop codon. The red sequence represents the upstream primer sequence and the blue sequence represents the downstream primer sequence. The black part indicates a similarity of 100%. [file 12870_2021_3199_MOESM6_ESM.docx]

**The Overexpression of Four *MiTFL1* Genes from Mango Delays the Flowering Time in Transgenic *Arabidopsis***

Yi-Han Wang*, Xin-Hua He*, Hai-Xia Yu, Xiao Mo, Yan Fan, Zhi-Yi Fan, Xiao-Jie Xie, Yuan Liu, Cong Luo**

*College of Agriculture, State Key Laboratory for Conservation and Utilization of Subtropical Agro-Bioresources, Guangxi University, Guangxi Nanning, 530004*

**These authors contributed equally to this work.*

***Corresponding author: Cong Luo, 22003luocong@163.com*

Supplement Figure 4

The primer design site of *MiTFL1s* gene was used for qRT-PCR.

cDNA sequence comparison of the four genes, where the yellow shaded part represents the start codon and the red shaded part represents the stop codon. The red sequence represents the upstream primer sequence and the blue sequence represents the downstream primer sequence. The black part indicates a similarity of 100%.

TFL1-3 ATGTCAAGAATG---TCGGAGCCACTTGCGGTGGGGAGAGTGGTTGGTGATGTGGTGGAC

TFL1-4 ---------ATG---GCGGAGCCACTGGCTGTAGGGAGAGTGGTGGGTGATGTTGTGGAC

TFL1-1 ATGGCAAGAAT---AGTAGACCCTCTTGTTGTGGGGAGAGTGATAGGAGATGTTCTTGAT

TFL1-2 ATGGCAAGAATGCCATCAGATCCTCTTGTAGTTGGCAGAGTGATTGGAGATGTTGTTGAT

** .** **:** * ** ** ******.* **:***** * **

TFL1-3 ATTTTCACCCCAAGTGTGAAGATGACTGTAACTTATAACCCCAATAAGCAAGTTGCCAAT

TFL1-4 AATTTCAGCCCCAGTGTGAAGATGACTGTGACTTATAGCTCCAATAAACAAGTCGCTAAT

TFL1-1 TCTTTCTCTCCAACAATCACAATG---TTTGTAAGTTACAATAACAGGCAAGTCTGTAAT

TFL1-2 TGTTGTGATCAAACTGTCAAAATGGCAGTCACCTACAACTCTTCCAAGCAGGTGTACAAT

: ** *..* :.* *..*** * . :. :.* . :. *..**.** ***

TFL1-3 GGTTATGAGCTTACTCCTGCTGTCATTGCTGCTAAACCCAGGGTTGAGATTGGTGGAGAA

TFL1-4 GGTTATGAGCTTATGCCTGCTGCCATTGCTGCTAAACCTCGGGTTGAGATTGGTGGCGAG

TFL1-1 GGCCATGAGCTTTTACCTTCCACAGTTTCCTTCAGACCTAGGGTTGAGATTCAAGGAGGT

TFL1-2 GGCCATGAGCTGTTTCCATCTTCAGTGACTGTGAAACCTAAGGTTGAGGTTCACGGAGGT

** ******* : **: * ..* * *.*** ..*******.** . **.*.

TFL1-3 GATATGAGAGCTGCCTACACATTGATCATGACTGACCCTGATGCTCCAAGCCCTAGTGAT

TFL1-4 GACTTGAGAGCTGCCTACACATTGATCATGACTGACCCTGATGCTCCAAGCCCTAGTGAT

TFL1-1 GATATGAGAACTTTCTTTACACTGGTGATGACAGACCCAGATGTTCCTGGACCTAGTGAT

TFL1-2 GATATGAGATCATTCTTCACATTGATCATGACAGACCCAGATGTTCCTGGTCCAAGTGAC

** :***** *: **: *** **.* *****:*****:**** ***:.* **:*****

TFL1-3 CCATACCTGAGAGAACATCTCCACTGGATGGTTACGGACATTCCTGGTACCACTGATGTT

TFL1-4 CCATGCATGAGAGAACATCTCCACTGGATGGTTACAGACATTCCTGGAACAACTGATGTT

TFL1-1 CCTTACTTAAGGGAGCACCTGCACTGGCTAGTGGCAAACATACCAGGAACAACAGATGTC

TFL1-2 CCATACTTGAGGGAGCATTTACACTGGGTGGTGACAGACATCCCAGGCACAACTGATGCC

**:*.* *.**.**.** * ****** *.** .*..**** **:** **.**:****

TFL1-3 TCCTTTGGAAAAGAAGTGGTAAGTTATGAGATTCCAAAGCCGGTGGTGGGCATCCACAGG

TFL1-4 TCCTTTGGAAAAGAAGCGGTGGGTTATGAGATTCCAAAGCCGGTGGTGGGCATCCATAGA

TFL1-1 ACATTTGGGAGGGAACTGGTGAGTTATGAGATACCAAGGCCAAATATAGGCATCCACAGA

TFL1-2 ACGTTTGGAAGGGAATTGGTGAACTATGAAATGCCGAGGCCAAATATTGGGATTCACAGG

:* *****.*..*** ***... *****.** **.*.***..: .* ** ** ** **.

TFL1-3 TACGTATTCATATTGTTCAAGCAAAGAGGAAGACAAACTGTG---AAGACACCAACTTCA

TFL1-4 TACGTATTCATATTGTTCAAACAAAGAGGAAGACAGACTGTG---AAGGCACCAACTTCA

TFL1-1 TTTGCTTTTGCTCTATTCAGGCAGAAAGGTAGACAGATATT---TAACCCACCTTCTTCA

TFL1-2 TTTGTTTTCCTTCTGTTCAGGCAGAAACGCAGGCAAACAGTGATAAGTATACCATCTTCA

*: * :** : *.****..**.*.* * **.**.* : * *. ***::*****

TFL1-3 AGGGACTATTTTAACACAAGGCGGTTCGCACAAGACAACGGCCTCGGCCTGCCAGTGGCT

TFL1-4 AGGGACCATTTCAACACAAGGCAGTTTGCACAGGACAACGGCCTTGGCCTGCCTGTGGCT

TFL1-1 AGGGATAACTTCAGCACTCGAATTTTCGCTGCTGAAAACGATCTCGGTCCTCCTGTTGCT

TFL1-2 AGGGACGGCTTCAACACAAGAAAGTTTGCTGAAGACAATGACCTTGGTCAGCCTGTTGCA

***** . ** *.***:.*.. ** **: . **.** *. ** ** * **:** **:

TFL1-3 GCAGTCTACTTCAATGCCCAGAGAGAAACGGCTGCCAGAAGAAGATGAAGACGAAGACAT

TFL1-4 GCGGTCTACTTTAATGCCCAGAGAGAAACTGCTGCAAGAAGAAGATGAAGAAAAA-----

TFL1-1 GCCGTTTACTTCAACGCACAAAGAGAAACTGCAGCTAGAAGACGCTAACCAGATCCCCAC

TFL1-2 GCTGTGTTCTTCAATGCACAAAGGGAAACAGCCGCAAGAAGGCGCTG-------------

** ** *:*** ** **.**.**.***** ** ** *****..*.*.

TFL1-3 AGTAGAGATATCAGTTTGATACTAATTCCCGAGAAATAAAAGAAAACTGCTCGACTTCAA

TFL1-4 ------AGAAACAGTTCAATACTGCTTCAGGCGAAATAAATAAAACTG--CCAACTTCAA

TFL1-1 ATCCGCCATTTTCGTCCAGTCTATTTGGAAACCAGAAAATAAAAAATTACCCATTTGTAA

TFL1-2 ----------------------------------ATAAATCAGGAAATAATTATAAAAAA

.::**: ...*. .: : **

TFL1-3 CTTGAAGTTGGCTGCATTTTCCTCTTGTTCATGTTTTTAGGGTTTTGAATCGAATATGGA

TFL1-4 CTTGAATTTGGCAG-------CTCTAGTTCTTGTTTTAGGGTCTTGGGTTCAAATGTCTG

TFL1-1 ATCTAAATAACATGCAG----TAATGGAGAAAATTACCATCTTTGTACCTGCAATTCCGC

TFL1-2 ATAAGGTACTATTCCAG----AAGGAAAAAATATTTAAAACATTTTAATTTGAAAAGGCG

.* .. : : : .: .::.**: . * . * **:
